# Structural basis of norepinephrine recognition and transport inhibition in neurotransmitter transporters

**DOI:** 10.1038/s41467-021-22385-9

**Published:** 2021-04-13

**Authors:** Shabareesh Pidathala, Aditya Kumar Mallela, Deepthi Joseph, Aravind Penmatsa

**Affiliations:** Molecular Biophysics Unit, Indian Institute of Science, Bangalore, India

**Keywords:** X-ray crystallography, Transporters in the nervous system

## Abstract

Norepinephrine is a biogenic amine neurotransmitter that has widespread effects on alertness, arousal and pain sensation. Consequently, blockers of norepinephrine uptake have served as vital tools to treat depression and chronic pain. Here, we employ the *Drosophila melanogaster* dopamine transporter as a surrogate for the norepinephrine transporter and determine X-ray structures of the transporter in its substrate-free and norepinephrine-bound forms. We also report structures of the transporter in complex with inhibitors of chronic pain including duloxetine, milnacipran and a synthetic opioid, tramadol. When compared to dopamine, we observe that norepinephrine binds in a different pose, in the vicinity of subsite C within the primary binding site. Our experiments reveal that this region is the binding site for chronic pain inhibitors and a determinant for norepinephrine-specific reuptake inhibition, thereby providing a paradigm for the design of specific inhibitors for catecholamine neurotransmitter transporters.

## Abstract (web summary)

The *Drosophila* dopamine transporter (dDAT) is a catecholamine neurotransmitter transporter that resembles the human norepinephrine transporter (hNET). Here the authors report X-ray structures of the dDAT in substrate-free form, norepinephrine-bound form and dDAT bound to commonly prescribed chronic pain inhibitors duloxetine, milnacipran and tramadol and shed light on the structural basis of norepinephrine recognition and transport inhibition in neurotransmitter transporters.

## Introduction

Neurotransmitter transporters of the solute carrier 6 (SLC6) family enforce spatiotemporal control of neurotransmitter levels in the synaptic space through Na^+^/Cl^−^-coupled uptake in the central and peripheral nervous systems^1–3^. Monoamine neurotransmitters affect diverse neurophysiological processes, including attention, arousal, sleep, mood, memory, reward, vasodilation, and pain^4–8^. Among monoamines, noradrenaline/norepinephrine (NE) is an important neurotransmitter released from the neurons of locus coeruleus in the brain stem that innervate multiple regions of the brain and spinal cord^9^. Discovered by vonEuler as a demethylated form of adrenaline^10^, NE was identified as a neurotransmitter with agonistic effects on the *α*- and *β*- adrenergic receptors^11^. The levels of biogenic amines, NE, dopamine (DA), and serotonin (5-HT), in the neural synapses, are controlled by their cognate transporters, NET, DAT, and SERT, respectively^1,12–15^. Recent structural studies of the *Drosophila* dopamine transporter (dDAT) and the human serotonin transporter (hSERT) reveal that the SLC6 members closely share their architecture and mechanistic properties^16–18^. The structural similarities among biogenic amine transporters extend to overlapping substrate specificities, particularly between DAT and NET, which are both capable of DA and NE uptake, albeit with varying efficacies^19^.

Biogenic amine transporters are the primary targets of antidepressants and psychostimulants that inhibit monoamine transport and enhance neurotransmitter levels in the synaptic space^20,21^. Specific inhibitors of monoamine uptake including selective serotonin reuptake inhibitors (SSRIs), serotonin-norepinephrine reuptake inhibitors (SNRIs), and norepinephrine reuptake inhibitors (NRIs) are widely used to treat depression in comparison with less-selective inhibitors of monoamine transport^22,23^. SNRIs and NRIs are also repositioned and prescribed as medication for chronic pain conditions including neuropathic pain and fibromyalgia^24^. They enhance NE levels in the descending pain pathways innervating the dorsal horn of the spinal cord. In this process, NE-mediated activation of the inhibitory *α*2-adrenergic receptors lead to lowered Ca^2+^-channel activation and promote hyperpolarization, to reduce chronic pain^5^. Most inhibitors of monoamine transport competitively inhibit uptake through interactions in the primary binding site^16,25,26^. In addition to interactions at the primary binding site, an allosteric binding site for citalopram that causes non-competitive inhibition of 5-HT transport is observed in the extracellular vestibule of hSERT^27^. This secondary site, in LeuT, a bacterial homolog of neurotransmitter transporters, displays interactions with detergent molecules^28,29^. Instances of non-competitive inhibition are increasingly observed in other neurotransmitter transporters like hDAT^30^ and human glycine transporters (hGlyTs)^31^.

**Fig. 1:**
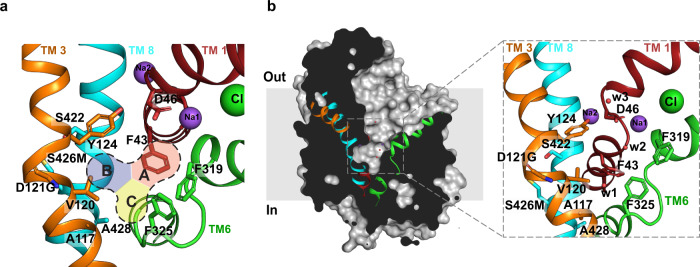
Organization of primary binding site and substrate-free dDAT. **a** Close-up view of substrate-free dDAT_subB_-binding pocket, showing the organization of subsites A, B, C of the primary binding site colored as red, blue, and yellow, respectively. **b** Surface representation of substrate-free dDAT_subB_ structure viewed parallel to the membrane plane. Helices TM1, TM3, TM6, and TM8 are colored as red, orange, green, and cyan, respectively. Inset shows the residues lining the primary binding pocket with water molecules indicated as red spheres.

The primary binding site of biogenic amine transporters is divided into subsites A, B, and C to delineate the regions of the molecule that interact with substrates and inhibitors^32,33^ (Fig. 1a). It is also observed that the primary binding site displays remarkable plasticity to accommodate inhibitors of varying sizes^26^. Alteration of the subsite B residues in dDAT to resemble hDAT or hNET yields a transporter with improved affinities to inhibitors including cocaine, *β*-CFT and the substrate analog 3,4-dichlorophenylethylamine (DCP)^26^. Similarly, the SSRIs also inhibit hSERT through interactions at the primary binding site^17^.

Despite recent progress in understanding the pharmacology and transport mechanism of neurotransmitter transporters through dDAT and hSERT structures, questions linger as to whether DA and NE, both catecholamines, have a similar mode of recognition in hNET. Given the lack of an experimental NET structure, it is also confounding as to how inhibitors can be designed with high specificity towards NET over DAT despite sequence identities >65%. In this context, X-ray structures of dDAT in complex with substrates, including DA, DCP, and d-amphetamine, have provided a glimpse into substrate recognition and consequent conformational changes that occur in biogenic amine transporters^26^. Incidentally, dDAT is also capable of NE transport similar to its mammalian orthologues and is well known to have greater affinities towards NE reuptake inhibitors^34,35^.

In this study, we employ dDAT as a surrogate of hNET to study the interaction of NE within the primary binding site. Comparison of different dDAT structures including the substrate-free, DA, and NE-bound states allow us to observe and explore interesting differences in substrate recognition in this transporter. Using X-ray structures of dDAT (Supplementary Table 1) in complex with popularly prescribed inhibitors of chronic pain including *S*-duloxetine, milnacipran, and a synthetic opioid, tramadol, we identify the importance of subsite C as the major determinant of inhibitor specificity between NET and DAT. We also validate these observations through hDAT-like mutagenesis in the subsite C region of dDAT that leads to a loss of affinity towards the NRIs used in the study.

## Results and discussion

### Modified dDAT resembles hNET primary substrate-binding site

**Fig. 2:**
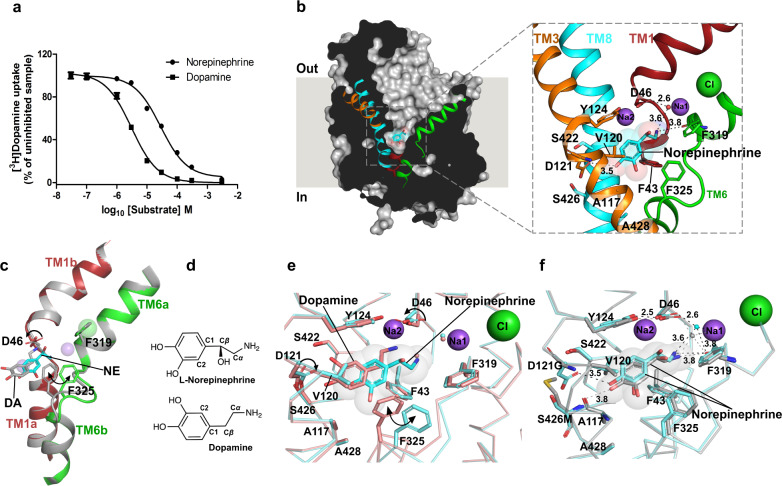
NE binds in a different pose in comparison with DA-bound dDAT. **a** Inhibition of [^3^H] dopamine uptake by dopamine and l-norepinephrine with inhibition constants (*K*_*i*_) of 2.0 ± 0.14 μM and 19.1 ± 1.7 μM, respectively. The data in the plot is a mean of *n* = 9 measurements obtained over three independent experiments and error bars representing s.e.m. **b** Longitudinal section of l-norepinephrine (NE)-dDAT_mfc_ complex with l-norepinephrine displayed as cyan spheres. Close-up view of the l-norepinephrine in the primary binding site with surrounding residues displayed as sticks. Hydrogen bond interactions are depicted as dashed lines. **c** An overlay of TMs 1 and 6 display no change in helix positions, but highlights a shift in the main chain of the TM6 linker (C*α* rmsd of 1.4 Å between residues 322 and 326) between the dopamine (DA) bound (gray) and norepinephrine (NE) bound structures (colored helices). **d** Chemical structures of l-norepinephrine and dopamine displayed to highlight differences in binding poses. **e** Comparison of binding pockets of NE-dDAT_mfc_ complex (NE in cyan and backbone in aquamarine) and DA-dDAT_mfc_ (PDB id. 4XP1) complex (DA in deep salmon and backbone in salmon). The D46 sidechain remains in a position similar to antidepressant-bound structures of dDAT (*χ*^1^ torsion angle +85°) unlike the dopamine bound structure (*χ*^1^ torsion angle −175°). The D121 (TM3) residue in subsite B also shifts (*χ*^2^ shifts by 16°) to interact with NE in comparison with the DA-bound dDAT structure. The position of F325 shifts by nearly 2 Å (C*β*) with a corresponding rotation of the phenyl group by 51° (*χ*^2^ torsion angle CD1-C*γ*−C*β*-C*α*). **f** Superposition of binding pockets of the NE-dDAT_mfc_ structure (NE and backbone in cyan) with the binding pocket of the NE-dDAT_NET_ structure carrying hNET-like mutations in subsite B (NE and backbone in gray). NE was modeled into densities at near identical positions in both the structures. Source data are provided as a Source Data file.

The dDAT, much like its human counterparts hDAT and hNET, is capable of interacting with both DA (*K*_*i*_ = 2.0 μM) and NE (*K*_*i*_ = 19.1 μM) with varying efficacies (Fig. 2a). The dDAT transports DA with a *K*_M_ of 3.6 μM (Supplementary Fig. 1a) and was proposed as a primordial catecholamine transporter in fruit flies^34^. A comparison of dDAT with hNET and hDAT reveals that SNRIs used in the study, duloxetine, milnacipran, and tramadol display inhibition potencies that are similar to hNET in comparison with hDAT (Supplementary Table 2). The amino-acid sequence of dDAT in the primary binding site has high similarity to hNET and hDAT (Supplementary Fig. 1b), and has pharmacological characteristics closer to hNET whilst having better transport characteristics with DA^34^. The dDAT primary binding site is identical to hNET in subsites A and C, whereas it differs by two residues in subsite B with polar substitutions; Asp, instead of Gly, at position 121 (149 in hNET) and Ser, instead of a Met, at position 426 (424 in hNET) (Supplementary Fig. 1b, c). Despite these differences, WT dDAT displays a nisoxetine-binding affinity (*K*_*d*_ = 5 nM) that is very close to hNET (*K*_*d*_ = 1.9 nM)^34,36^. The dissociation constants (*K*_*d*_) measured for the dDAT constructs, used in this study, also display high-affinity interactions in the range of 2.8–3.8 nM for nisoxetine, similar to hNET (Supplementary Table 2). Besides these, a vestibule-lining phenylalanine in dDAT was mutated to its hNET counterpart leucine (F471L) to make it resemble hNET. The presence of leucine at this site was reported to be important for the specific inhibition of hNET by the *χ*-conotoxin, MrIA^36^. We investigated the effects of substituting these amino acids in the subsite B and vestibule of dDAT on its transport activity. The vestibular mutation F471L did not result in a significant loss of transport activity (~7%) in comparison with a functional construct of dDAT (dDAT_fc_). However, introducing D121G substitution led to a 30% reduction in the transport activity, and the S426M mutation resulted in a functionally inactive transporter (Supplementary Fig. 1d). The effect of the two residues on transport activity suggests that the residues in subsite B play a crucial but as yet unidentified role in the transport activity of dDAT.

Despite the inability of dDAT with these mutations in subsite B to transport catecholamines, it is used in this study as it reproduces the binding site of hNET, along with the F471L substitution in the vestibule. This construct, hereafter, referred to as dDAT_NET_ closely resembles the binding propensities of monoamine transport inhibitors with hNET than with hDAT (Supplementary Table 2). The structure of dDAT_NET_ construct was obtained in its substrate-free, NE-bound, and inhibitor-bound forms. Besides this, a functionally active construct (dDAT_mfc_) (Supplementary Fig. 1d) was also used to obtain a structure complexed with NE. All the crystal structures were obtained in complex with a heterologously expressed, synthetic version of 9D5 antibody fragment (Fab) that was previously used to crystallize the dDAT^16,26,35^ (Supplementary Fig. 2).

### Substrate-free state is outward-open

The dDAT_subB_ construct, with hNET-like mutations, was crystallized in the substrate-free Na^+^ and Cl^−^-bound conformation to observe for structural changes in the binding pocket. The transporter structure, determined at 3.3 Å resolution, displays an outward-open conformation with a solvent-accessible vestibule that is largely devoid of any specifically bound moieties except for the Na^+^ and Cl^−^ ions at their respective sites (Fig. 1b). Despite the absence of bound substrate or inhibitor in the primary binding site, multiple blobs of positive density were observed within the extracellular vestibule into which a polyethylene glycol (PEG) was modeled (Supplementary Fig. 3a). Incidentally, the position of PEG coincides with the position of the detergent binding secondary site in LeuT and also with *S*-citalopram bound allosteric site in hSERT (Supplementary Fig. 3b, c). Within the primary binding site, clear density was observed for most of the residues lining the binding pocket. Solvent accessibility into the primary binding site is unhindered by the F319, which remains splayed open thereby retaining dDAT in an outward-open conformation (Fig. 1b). Interestingly, the sidechain of F325 located in the TM6 linker is positioned in a manner that resembles the antidepressant-bound conformation resulting in a primary binding site with substantial solvent accessibility (Supplementary Fig. 4). A positive density in the vicinity (∼3.1 Å) of F325 was observed into which a water molecule was positioned, allowing the F325 to have lone pair-π interactions (Fig. 1b). The outward-open conformation of the dDAT substrate-free state is consistent with the behavior of other NSS members including LeuT whose substrate-free ion-bound conformation is also in the outward-open state^37,38^. The addition of Na^+^ to LeuT induces the opening of the extracellular vestibule, suggesting the formation of an outward-open state, which is altered upon interactions with substrates like alanine or leucine that induce an occluded state^39^. Recent HDX measurements on dDAT and hSERT have clearly indicated the presence of an outward-open conformation in their ion-bound substrate-free states^40,41^. Similar observations were evident in all-atom simulations performed on a hDAT model built using dDAT as a template^42^. The demonstration of an outward-open conformation in the crystal structure of the substrate-free form of dDAT is a corroboration of these biophysical and computational observations. However, the presence of extraneous factors including a non-specifically bound PEG molecule in the vestibule and the propensity of the antibody fragment to bind an outward-open conformation of the transporter could further aid in stabilizing the substrate-free transporter in an outward-open state.

### NE binds in a different pose in comparison with DA

The structural similarity between DA and NE allows them to act as dual substrates for both the NET and DAT orthologues. While the hNET is capable of transporting DA and NE with *K*_*M*_ values of 0.67 μM and 2.6 μM, respectively, the hDAT can transport DA and NE with *K*_*M*_ values of 2.54 μM and 20 μM, respectively^19^. It is consistently observed that both NET and DAT orthologues preferably interact with DA with a higher affinity than NE. It is also observed that in DAT-knockout mice, NET can transport DA and substitute for its absence^43^. The DA uptake through dDAT can be competed by DA and NE with *K*_*i*_ values of 2.0 μM and 19 μM, respectively (Fig. 2a). These observations validate the use of dDAT as a useful substitute for hNET to study NE interactions.

The NE-bound dDAT_mfc_ structure reveals clear density for NE bound within the primary binding site of the transporter (Fig. 2b, Supplementary Fig. 5a, b). The primary amine of NE interacts in the subsite A region forming hydrogen bonds with carbonyl oxygens of F43 and F319 main chain and the carboxylate sidechain of D46 via a water molecule (Fig. 2b). The D46 residue in the DA-bound dDAT structure undergoes a *χ*^1^ torsion angle shift of 100° relative to that of the NE-bound structure to interact with the primary amine of DA. However, no such shift was observed in the NE-bound dDAT structure (Fig. 2c). The primary amine interacts with a Na^+^-coordinating water molecule akin to the d-amphetamine and DCP-bound structures (Supplementary Fig. 6). Interestingly, the binding pose of NE does not resemble that of DA in the binding pocket despite both the substrates being catecholamines (Fig. 2c–e). Earlier computational studies predicted that the catechol group of NE predominantly interacts with subsite B region^44,45^. However, we observe that the NE catechol group binds in the vicinity of subsite C in the region between TM6 linker and TM3 with no conformational changes in the binding pocket in comparison with the outward-open substrate-free form of dDAT (Fig. 2b, Supplementary Fig. 6a). The binding of NE in the primary binding site resembles a lock-and-key association in comparison to the induced-fit interaction observed with DA binding. Clear density for the *β*-OH group of NE is observed in the primary binding site (Supplementary Fig. 5a, b) and the *β*-OH group faces the solvent-accessible vestibule. The para-OH group of the catechol ring retains interactions with the sidechain carboxyl of D121 that undergoes a rotation of 16° along the *χ*^2^ torsion angle in comparison with the DA-bound structure, to facilitate interactions with NE (Fig. 2e). The meta-OH group of NE displaces the water molecule observed in the substrate-free state, fitting snugly into the gap between A117 and TM6 linker adjacent to the F325 sidechain (Supplementary Fig. 6a). DA, on the other hand, interacts closely with residues in subsite B, displaying a ~180° flip in the position of the catechol group, relative to NE (Fig. 2d, e). This induces a shift in the position of the disordered region of the TM6 linker where the main-chain shifts by a C*α* root-mean-square deviation of 1.43 Å for residues 322–326 in the DA-bound structure. This stretch of residues includes a Gly-Pro-Gly motif with the G324 displaying the maximal C*α* deviation of 2.4 Å, allowing the F325 (C*α* shift of 1.6 Å) to facilitate edge-to-face aromatic interactions with DA, which are absent in the NE-bound structure (Fig. 2c, e, Supplementary Movie. 1). Shifts in the TM6 linker have been observed before and are vital to remodel the binding site in response to substrate and inhibitor interactions^26,46^.

The subsite B residues in dDAT differ from hNET and hDAT at two positions, D121G (TM3) and S426M (TM8) (Supplementary Fig. 1b, c). In order to evaluate whether hNET-like substitutions in the subsite B of dDAT would influence and shift the conformation of NE to DA-like pose, we crystallized NE in complex with dDAT_NET_ construct that has D121G and S426M substitutions, alongside F471L. Despite hNET-like substitutions, no major shifts in the position of NE were observed in the binding site. Despite absence of the D121 sidechain, the para-OH group of NE establishes interactions with the main-chain carbonyl oxygen of A117 (Fig. 2f).

The comparison between DA and NE-bound crystal structures displays an unexpected difference between preferred conformers of DA and NE within the binding pocket. The catechol ring of NE is positioned in opposite orientation relative to that of DA with a minor difference in the C_2_C_1_-C_*β*_C_*α*_ torsion angle (26°) (Fig. 2d). This difference in their binding poses, despite being very similar catecholamines, can be attributed to the presence of *β*-OH group in NE, which restricts the rotation of its catechol ring along the C_1_–C_*β*_ bond with an energy barrier of 9–12 kcal/mol^47^. In the case of DA, the energy barrier for the same rotation is as low as 0.3–0.6 kcal/mol^48^. The low energy barrier for the rotation of the catechol group in DA allows greater conformational sampling and enables it to interact in close proximity to the subsite B of the primary binding site. On the other hand, the energy barrier for catechol rotation in NE, owing to the *β*-OH group, restricts its pose such that it is oriented more towards subsite C relative to DA and results in a relatively weaker interaction with the transporter. The binding of DA to dDAT results in an inward movement of the non-helical linker between TM6a-TM6b by 1.43 Å relative to that in the NE-bound state (Supplementary Movie 1). The induced-fit conformational change in response to DA binding likely leads to larger binding free energy changes, primarily caused by conformational changes and the formation of non-covalent interactions within the binding site yielding larger enthalpy differences, upon binding^49^. In the case of NE, interaction in the binding site results in the displacement of water with no consequent conformational changes, therefore resembling a lock-and-key association. We can therefore infer that the difference in flexibility of the two catecholamines, caused by the *β*-OH group, translates to both DAT and NET to have a greater propensity to interact with DA in comparison to NE.

### S-duloxetine and milnacipran are competitive inhibitors of NE transport

**Fig. 3:**
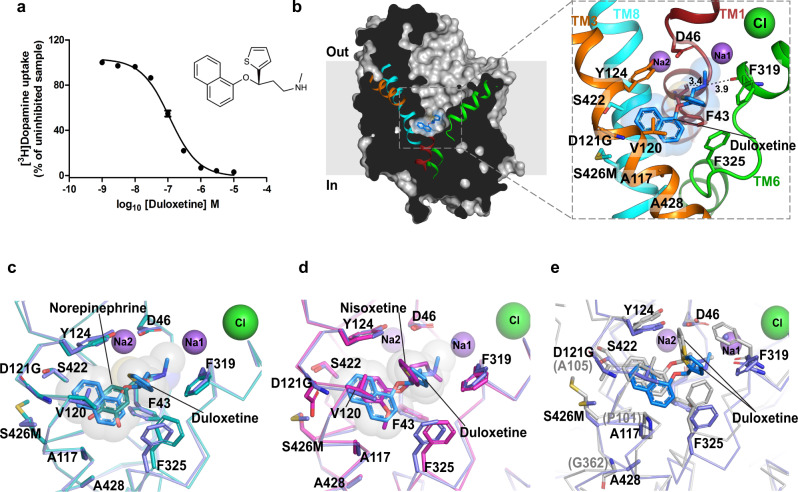
*S*-duloxetine binds in the primary binding site. **a** Inhibition of [^3^H] dopamine uptake with *S*-duloxetine with a *K*_*i*_ of 69.4 ± 2.9 nM (data in the plot is a mean of *n* = 9 measurements obtained over three independent experiments and error bars representing s.e.m). **b** Surface representation of dDAT_subB_ with bound *S*-duloxetine shown as blue sticks and transparent spheres in the primary binding site. Inset shows orientation of the inhibitor in the binding pocket with residues in the vicinity represented as sticks. **c** Binding site comparison of *S*-duloxetine with NE-bound dDAT_NET_. The phenyl group of F325 shifts by 40° to retain edge-to-face aromatic interactions with the naphthalene group of *S*-duloxetine. **d** Overlay of the dDAT bound to *S*-duloxetine and nisoxetine (magenta) (PDB id. 4XNU). The surrounding residues display an identical binding pose between the two structures. **e** Overlay of duloxetine-bound dDAT_subB_ and LeuBAT structures (gray) (PDB id. 4MMD) displaying similar pose of the inhibitor in both the structures. LeuBAT displays a prominent occluded conformation, whereas dDAT retains an outward-open conformation. Source data are provided as a Source Data file.

**Fig. 4:**
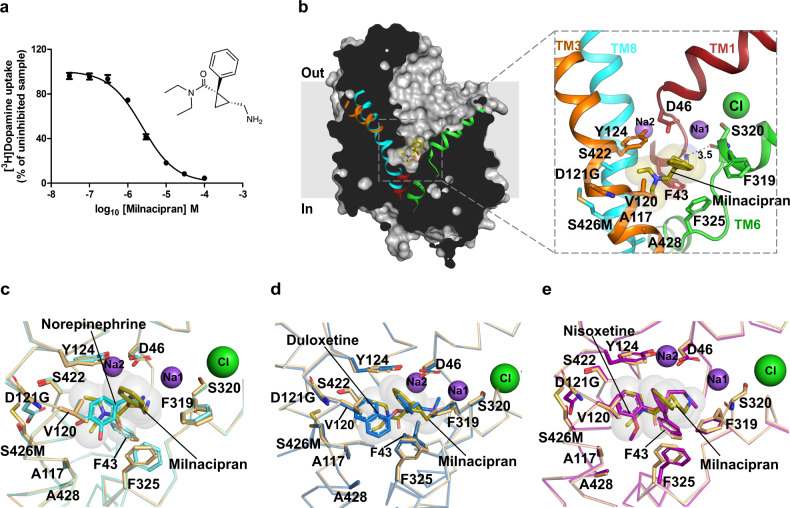
Milnacipran is a competitive inhibitor of NE uptake. **a** [^3^H] dopamine transport inhibition by increasing concentrations of 1R-2S milnacipran with a *K*_*i*_ value of 1.5 ± 0.1 μM (data in the plot are a mean of *n* = 9 measurements obtained over three independent experiments and error bars representing s.e.m). Inset displays the chemical structure of 1R-2S milnacipran. **b** Longitudinal section of dDAT_NET_ in complex with milnacipran-bound (olive sticks) in the primary binding site with residues in proximity displayed as sticks. **c** Milnacipran-binding pose compared with NE binding in dDAT_mfc_ reveals an angular shift of F325 by 35° to retain hydrophobic interactions with the diethyl group of milnacipran. **d** Milnacipran-bound dDAT_NET_ vs duloxetine-bound dDAT_subB_ display identical positions of the surrounding residues. **e** Structural overlap of nisoxetine bound dDAT (PDB id. 4XNU) with milnacipran-bound dDAT_subB_, revealing the surrounding residues in identical binding positions. Source data are provided as a Source Data file.

The ability of SNRIs to alleviate chronic pain by blocking NET activity in the descending pain pathways has allowed drugs like *S*-duloxetine and milnacipran to be repositioned for treatment of neuropathic pain and fibromyalgia^50^. Much like the other inhibitors/antidepressants characterized including nortriptyline, nisoxetine, and reboxetine in complex with dDAT^16,35^ and paroxetine, *S*-citalopram, fluoxetine in complex with hSERT^51^, both *S*-duloxetine and milnacipran interact at the primary binding site of the dDAT structure (Figs. 3, 4). The electron densities for both drugs are unambiguous and conform to the general principles of inhibitor interactions with the transporter (Supplementary Fig. 5c, d).

S-duloxetine, owing to its large surface area (surface area 505.7 Å^2^) exhibits maximal occupancy of the primary binding pocket. The drug inhibits DA uptake with a *K*_*i*_ value of 69.4 ± 2.9 nM (Fig. 3a), consistent with the *K*_*i*_ values observed for hNET inhibition by duloxetine^32^. The high affinity is an outcome of its ability to snugly fit into the cavernous primary binding site of NSS transporters. In duloxetine, the propanamine group interacts with the main-chain carbonyl oxygens from residues F43 and F319 with the D46 residue in the vicinity (Fig. 3b). The secondary amine can also mediate π–cation interaction with the sidechain of F43. The naphthyloxy ring interactions in the binding pocket extend from subsite B to subsite C, wedging into space sculpted by residues including Y124, D121G, S426M, V120, A117 in TM3 and TM8 followed by edge-to-face aromatic interactions with F325 in subsite C. The thiophene ring is positioned with some elevation within the binding pocket to sterically block the closure of the F319 and thus precluding the formation of an occluded state during transport (Fig. 3b). The duloxetine position in the binding pocket overlaps with NE and nisoxetine binding to a large extent with the naphthalene ring taking the place of the methoxyphenyl ring of nisoxetine, a specific inhibitor of NE reuptake (Fig. 3c, d). Similarly, when compared with the cocaine-bound structure of dDAT, one of the aromatic groups of the naphthalene ring in duloxetine overlaps with the benzoyl moiety of cocaine (Supplementary Fig. 7). However, the lack of an additional hydrophobic moiety in cocaine makes it a moderate inhibitor of NE uptake, relative to duloxetine^52^. The position of duloxetine in the binding pocket is very similar to the LeuBAT-duloxetine complex elucidated earlier, thus corroborating LeuBAT as a relevant model system to study the pharmacology of biogenic amine transporters (Fig. 3e)^33^.

Milnacipran has an unconventional structure with a cyclopropyl skeleton having both a primary amine and tertiary amine (*N*, *N*-diethyl) being part of the drug structure^53^. The drug lacks large aromatic moieties that are commonly observed with most NSS inhibitors. Milnacipran inhibits DA transport by dDAT with a *K*_*i*_ value of 1.5 ± 0.1 µM, which is much higher than *S*-duloxetine (Fig. 4a). Like duloxetine, milnacipran also binds in the primary binding site and overlays well with NE (Fig. 4b, c). The primary amine of aminomethyl group in milnacipran interacts with the sidechain of D46 and the main-chain carbonyls of F43 and S320 in the subsite A by hydrogen bonds (Fig. 4b). Altering the hydrophobicity of the binding site by substituting residue V148 (V120 in dDAT_NET_) in hNET to an isoleucine leads to a 17-fold enhancement of milnacipran’s inhibitory potency^32^. The phenyl group attached to the chiral center at the cyclopropane group overlaps with the thiophene group of duloxetine and phenyl ring of nisoxetine (Fig. 4d, e). Interestingly, the *N*, *N*-diethyl group, which is usually occupied by bulky aromatic groups in most of the inhibitors, does not wedge deeply into the subsite B as observed with cocaine (Supplementary Fig. 7c) and retains hydrophobic interactions in the vicinity of subsite C. The absence of a bulky aromatic group wedging into subsite B could be the reason for the lowered transport inhibition observed in milnacipran in comparison to duloxetine (Supplementary Table 2).

### Synthetic opioid tramadol blocks transport by interacting with subsite C

**Fig. 5:**
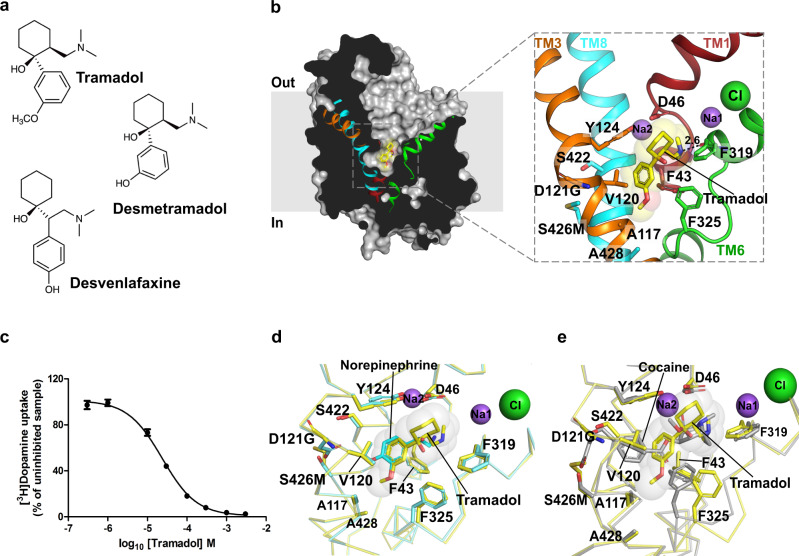
Tramadol is a synthetic opioid that inhibits NE uptake. **a** Chemical structures of inhibitors, tramadol, desmetramadol, and desvenlafaxine. **b** Structure of tramadol (yellow) bound dDAT_NET_ viewed parallel to the membrane plane where tramadol was observed in the primary binding site. **c** [^3^H] dopamine uptake inhibition observed with increasing concentrations of tramadol displaying a *K*_*i*_ value of 15.9 ± 1.8 μM (data in the plot is a mean of *n* = 9 measurements obtained over three independent experiments and error bars representing s.e.m). **d** Structural comparison around the primary binding site between tramadol bound dDAT_NET_ and NE-bound dDAT_mfc_ wherein the methoxyphenyl group of tramadol overlaps exactly with the catechol ring of NE. No differences in the positions of the binding site residues were observed. **e** Comparison of tramadol-bound dDAT_NET_ structure with cocaine-bound dDAT_mfc_ structure (gray) (PDB id. 4XP4). The benzoyl ester group of cocaine clearly interacts with subsite B, whereas tramadol’s aromatic group interacts primarily at subsite C, within the primary binding site. Source data are provided as a Source Data file.

It is well known that some synthetic opioids have a dual mechanism of action for pain relief by serving as agonists of *μ*-opioid receptors and blockers of NE and 5-HT uptake. Tramadol is a popularly used synthetic opioid with a dual ability to activate opioid and NE-based analgesic pathways^54^. Although tramadol is considered a safe drug, it induces opioid-like symptoms and dependence when used in supra-therapeutic doses^55^. The demethylated metabolite of tramadol, o-desmethyl tramadol (desmetramadol) is a better agonist for opioid receptor whilst tramadol, particularly S,S-tramadol is specific to NE transport inhibition^56^. Tramadol and desmetramadol structurally resemble the antidepressant, desvenlafaxine (Fig. 5a). Tramadol inhibits the transport activity of dDAT with a *K*_*i*_ of 15.9 ± 1.8 µM (Fig. 5), which is substantially higher than that of *S*-duloxetine and milnacipran, indicating weaker affinity. Similarly, tramadol has higher *K*_*i*_ value of 5.3 ± 0.4 μM relative to the other studied inhibitors for competitively displacing nisoxetine from the binding pocket (Supplementary Fig. 8). Despite weaker affinities to the transporter, tramadol is highly specific to NET/SERT over DAT displaying ~50-fold greater affinity to NET over DAT^56^. The structure of the tramadol–dDAT_NET_ complex reveals that the drug binds to the primary binding site with the tertiary amine of the dimethylamino group interacting with subsite A residues D46 and carbonyl oxygens of F43 and F319 (Fig. 5b, Supplementary Fig. 5e). The 1-cyclohexanol group takes a similar position to the thiophene group of *S*-duloxetine and phenyl groups of milnacipran and nisoxetine to sterically prevent the formation of an outward-occluded conformation in the transport process (Supplementary Fig. 4). The lower affinity of tramadol to inhibit neurotransmitter uptake compared with other SNRIs could be attributed to the lack of an aromatic moiety in the close vicinity of the subsite B. This distinction is even more apparent when the tramadol bound dDAT_NET_ structure is compared with cocaine-bound dDAT_mfc_ structure where the benzoyl group of cocaine is clearly wedged into subsite B in comparison with tramadol’s methoxyphenyl ring that is primarily in the vicinity of subsite C (Fig. 5e). The methoxyphenyl ring interacts with the sidechain of Y124 by aromatic edge-to-face interactions and fits into the hydrophobic pocket lined by the side-chains of A117, V120, A428, and F325 of subsite C. The position of methoxyphenyl group coincides very closely with the catechol group of NE and methoxy group overlaps well with the meta-OH of NE (Fig. 5d). Much like nisoxetine and reboxetine, the methoxy group of tramadol occupies the space between A117 and F325. Interestingly, the demethylated metabolite of tramadol, desmetramadol is a weaker inhibitor of hNET (IC_50_ = 6 μM) relative to tramadol (IC_50_ = 2 μM)^56^. This indicates the importance of this hydrophobic interaction in enhancing the efficacy of selective NE reuptake inhibition. A close examination of the binding pocket reveals that hDAT and hNET have subtle differences within them. The dDAT, although largely resembling hNET in the binding pocket, differs from hDAT in the residues A117(TM3), S422(TM8), and A428(TM8). A117 and A428 residues line subsite C in the vicinity of the TM6 linker where NET-specific drugs like nisoxetine, reboxetine, milnacipran, and tramadol interact. The lack of interactions in subsite B and the presence of substitutions in subsite C (A117S and A428S) unique to human DAT, makes tramadol a weak hDAT inhibitor with a IC_50_ of ∼100 μM^56^. Tramadol exhibits similar *K*_*i*_ values for displacing nisoxetine bound to dDAT_mfc_ and dDAT_NET_ proteins wherein dDAT_NET_ has hNET-like D121G/S426M substitutions in subsite B (Supplementary Fig. 8). In contrast, inhibitors like cocaine, which primarily bind to subsite B have a 10-fold increment in their ability to compete for nisoxetine in the presence of hNET-like mutations in subsite B^26^. The observations from tramadol-dDAT complex clearly indicate that NET-specific inhibition occurs through interactions at subsite C. It is also evident that the lack of interactions at subsite B compromises the affinity of tramadol, but clearly fails to influence the specificity of NET inhibition in comparison with DAT^56^.

### Ligand binding to subsite C influences specificity of NE uptake inhibition

**Fig. 6:**
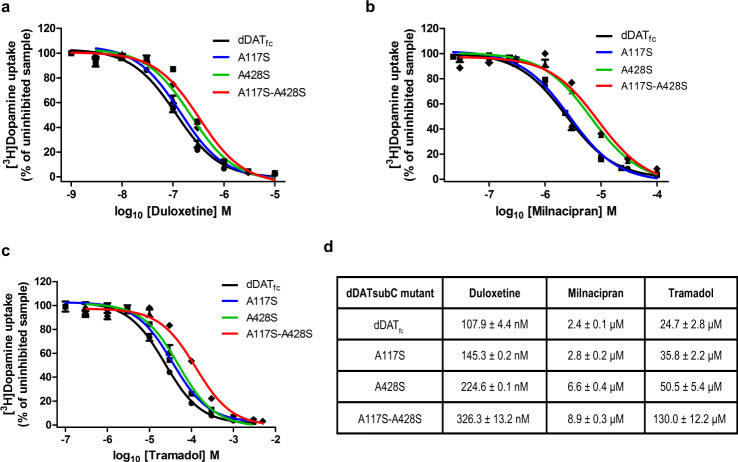
hDAT-like mutagenesis of residues leads to reduced affinities of NET-specific inhibitors. **a** Dose–response curves of [^3^H] dopamine uptake inhibition by duloxetine with a functional construct of dDAT carrying hDAT-like mutations in the primary binding site including dDAT functional construct (dDAT_fc_) (black), A117S (blue), A428S (green), and A117S/A428S double mutant (red). The uptake inhibition curves of subsite C mutants were compared with that of the dDAT_fc_ (black) plots shown in previous figures. **b**, **c** Similar uptake inhibition curves plotted using milnacipran and tramadol, respectively. Experimental plots for all mutants are means of *n* = 9 measurements obtained over three independent experiments and error bars representing s.e.m. **d** Table indicates IC_50_ values ± s.e.m of individual mutant inhibition curves with all three inhibitors. Substitution of subsite C residues A428S and double mutant A117S and A428S display maximal loss of affinity for SNRIs duloxetine, milnacipran, and tramadol. The loss in inhibition potencies is significant (*p* values for duloxetine, milnacipran, and tramadol are 2.44 × 10^−8^, 6.03 × 10^−10^, and 1.43 × 10^−5^, respectively) between the dDAT_fc_ and the dDAT A117S-A428S mutant. A two-tailed *t* test assuming unequal variances was performed to obtain the *p* values. Source data are provided as a Source Data file.

**Fig. 7:**
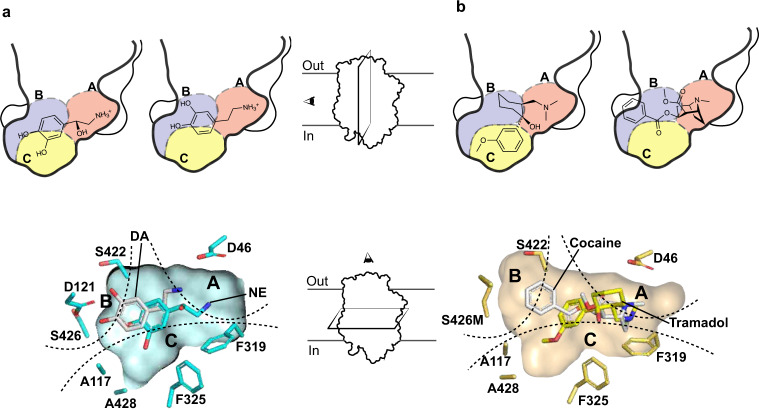
Distinct binding poses of substrates and NET inhibitors. **a** Lateral sections of the primary binding pocket comparisons between NE and DA-bound states reveal distinct conformations and binding poses of the two substrates in the subsite C and subsite B, respectively (top). Transverse section of the binding pocket clearly reveals similar distinctions between NE and DA (bottom). **b** Lateral section comparisons between tramadol and cocaine, displaying primary interactions of their aromatic moieties in subsite C and subsite B, respectively (top). Transverse section of the binding pocket showing tramadol overlapped with cocaine indicates clear differences in interaction of a non-specific inhibitor, cocaine towards subsite B and an SNRI, tramadol interacting preferentially with subsite C.

The structures of SNRIs duloxetine, milnacipran, and tramadol in complex with the dDAT show a progressively smaller aromatic moiety that interacts with the subsite B and C in the primary binding pocket of dDAT. In an earlier study, it was observed that the aromatic moieties in drugs like cocaine, RTI-55, and nisoxetine interact closely in the subsite B and have enhanced affinities when hNET-like substitutions D121G and S426M are made in the pocket^26^. Interestingly, this improvement in affinity is not apparent in the case of SNRIs employed in the study where the *K*_*i*_ values remain unchanged or weaken when substitutions in subsite B are made to improve the identity of dDAT-binding pocket to hNET (Supplementary Fig. 8). The minimal effect of subsite B substitutions on the affinity of duloxetine, milnacipran, and tramadol suggest that determinants of NET specificity lie elsewhere in the binding pocket. Earlier studies have posited that non-conserved residues in the primary binding site are responsible for selective inhibition of biogenic amine uptake^57,58^. In order to evaluate the role of the subsite C residues that differ between hNET and hDAT in the binding site, hDAT-like mutations A117S, A428S and a combination of A117S/A428S were introduced into *dDAT*_*fc*_. Effects of these mutations were analyzed through uptake inhibition using the three SNRIs employed in this study (Fig. 6a–c). Individual substitution of A428S at subsite C caused a marginal (two–threefold) loss of uptake inhibition, whereas the A117S mutation did not cause any significant change (Fig. 6d). However, a combination of A428S and A117S caused a substantial loss in the ability of duloxetine, milnacipran, and tramadol to inhibit DA transport as observed by a ~3, ~3.8, and ~5-fold increase in their IC_50_ values, respectively (Fig. 6d). The IC_50_ value for tramadol obtained with the A117S-A428S double mutant is very similar to its reported IC_50_ value with hDAT (Fig. 6d; Supplementary Table 2)^56,59^. Thus, these substitutions clearly indicate the importance of subsite C region in dictating the specificity of individual inhibitors that exploit this hydrophobic cavity to gain NET specificity. The polar substitutions observed in this region in hDAT lead to a reduced ability of SNRIs with hydrophobic moieties to bind efficiently, thereby compromising their ability to interact with the hDAT (Fig. 7a, b).

In conclusion, using X-ray structures of dDAT in complex with NE and NET-specific inhibitors, highlights the discrepancies in catecholamine recognition in neurotransmitter transporters and explores the basis for NET-specific reuptake inhibition over DA reuptake inhibition, despite the close similarity between NET and DAT. The catechol group of NE is observed to interact primarily at subsite C in the vicinity of NET-specific residues A117 and A428. The binding of NE displaces water molecules in the binding pocket observed in the substrate-free state and does not induce any local conformational changes in the binding, contrary to DA (Fig. 7a). DA was previously observed to interact closely with its catechol group in the vicinity of subsite B, leading to shifts in the positions of residues D46 and F325 to retain interactions with the neurotransmitter^26^. The absence of these changes in NE-dDAT complex could be attributed to the reduced flexibility of NE, in comparison with DA, owing to the presence of the *β*-OH group in its structure^47^.

The NET-specific chronic pain inhibitors, duloxetine, milnacipran, and tramadol, compete for NE-binding site through aromatic groups. These aromatic moieties can interact and snugly fit at subsites B and C to retain high affinity and selectivity towards specific biogenic amine transporters. However, hDAT-like mutations within subsite C of dDAT, A117S, and A428S alter the polarity of the binding pocket and weaken the hydrophobic interactions and prevent functional groups like the methoxyphenyl group of tramadol from accessing the subsite through steric block (Fig. 7b). On the other hand, non-specific inhibitors of biogenic amine transport, for instance cocaine, primarily interact with subsite B wherein hNET-like mutations D121G and S426M in dDAT enhance cocaine affinity by 10-fold. The primary interactions of cocaine at subsite B induces a plastic reorganization of the binding pocket as the subsite C residue F325 compensates for lack of bulky aromatic group in cocaine through local conformational changes to establish aromatic π-stacking interactions^26^. Through our results, we infer that NET-specific inhibitors could be designed with primary interactions at subsite C whilst non-specific high-affinity interactions are observed in inhibitors that interact at subsite B. Taken together, the results of this work convey the unique facets of catecholamine recognition within the same binding pocket and establish the roles of individual subsites in dictating inhibitor selectivity and affinity among biogenic amine transporters. The findings can effectively be used for selective inhibitor design targeting pharmacological niches as widespread as depression and chronic pain.

## Methods

### List of constructs

The *Drosophila melanogaster* DA transporter construct used for performing transport assays (*dDAT*_*fc*_) has a deletion of 20 amino acids in the amino-terminal (Δ1–20) and a deletion in the extracellular loop 2 (EL2) from 164 to 191 amino acids. It also contains two thermostabilizing mutations V74A, L415A, and F471L mutation in the vestibule to resemble human norepinephrine transporter (hNET). Additional mutations in the subsite B or subsite C were incorporated into this gene used for carrying out the uptake assays.

The uptake active *dDAT* construct used for co-crystallizing with NE (*dDAT*_*mfc*_) has amino-acid deletions Δ1–20 and Δ162–202 along with the two thermostabilizing mutations (V74A and L415A). A thrombin site (LVPRGS) insertion replaces residues 602–607 towards the C-terminus. It also has the F471L mutation in the vestibule (Supplementary Table 3).

*dDAT*_*subB*_ has deletions Δ1–20 and Δ162–202; thermostabilizing mutations V74A, L415A along with two mutations in subsite B of the substrate-binding pocket D121G and S426M (Supplementary Table 3). A thrombin site insertion, identical to *dDAT*_*mfc*_ is present in the C-terminus. This construct was used to elucidate the crystal structures of substrate-free and the duloxetine bound form.

*dDAT*_*NET*_ is identical to *dDAT*_*subB*_ with the additional mutation F471L in the vestibule. This construct was used to decipher the crystal structures in NE, milnacipran, and tramadol bound complexes of dDAT.

### Expression and purification of the transporter

The recombinant expression of *dDAT* constructs in pEG-BacMam vector was done using baculovirus-mediated protein expression in mammalian cells, where HEK293S GnTI^−^ cells were transduced with high titer recombinant baculovirus using the BacMam method^60^. The expressed dDAT protein was extracted from membranes in 20 mM dodecyl maltoside (DDM) (Anatrace), 4 mM cholesteryl hemisuccinate (CHS) (Anatrace), 50 mM Tris-Cl (pH 8.0), and 150 mM NaCl. The solubilized material was centrifuged at 100,000 × *g* to pellet down the unsolubilized material. The solubilized protein was affinity-purified using Talon resin (Takara Bio) in 20 mM Tris-Cl (pH 8.0), 300 mM NaCl, 100 mM imidazole containing 1 mM DDM, and 0.2 mM CHS. The affinity-purified protein was treated with thrombin (Haematologic Technologies Inc) for removing the C-terminal GFP-8x-His tag. The thrombin cleaved protein was purified by size-exclusion chromatography on a superdex-200 10/300 increase column (GE Life Sciences) in 4 mM decyl β d-maltoside (Anatrace), 0.3 mM CHS, 0.001% (w/v) 1-pamitoyl-2-oleoyl-*sn*-glycero-3-phospho ethanolamine (Avanti Polar Lipids), 20 mM Tris-Cl (pH 8.0), 300 mM NaCl, and 5% glycerol. The peak fractions were collected and pooled before incubating with the substrate (6 mM) or inhibitor (0.5–2.0 mM) to the indicated final concentrations used for crystallization. For complexes containing NE (Sigma Aldrich), 4 mM ascorbic acid (Sigma Aldrich) was added to prevent its oxidation.

### Heterologous expression and purification of Fab

The heavy and light chain genes of the *Fab 9D5* were synthesized (Genscript) and cloned into pFastBac Dual vector with the heavy chain under the polyhedrin promoter and the light chain under the P10 promoter with an N-terminal GP64 signal peptide on each chain. A TEV protease site followed by 8x-His tag was added to the C-terminus of heavy chain. The cloned plasmid was transformed into *Escherichia coli* DH10Bac (Thermo Fischer Scientific) for the generation of recombinant bacmids. The bacmids were then transfected into Sf9 cells (Invitrogen) to generate recombinant baculovirus. High titer recombinant virus was used to infect large volume Sf9 cell cultures and 96 h post infection, the cells were spun down and the supernatant containing Fab was dialyzed against 25 mM Tris-Cl (pH 8.0) and 50 mM NaCl. The dialysate was then passed through Ni-NTA beads (Qiagen), washed with 50 mM imidazole and eluted in 25 mM Tris-Cl (pH 8.0), 50 mM NaCl containing 250 mM imidazole. The eluted Fab was further purified by size-exclusion chromatography in 25 mM Tris-Cl (pH 8.0) and 50 mM NaCl using a superdex 75 10/30 column (GE Life Sciences). The purified Fab was stored at 4 °C.

### Crystallization and structure determination

The SEC-purified dDAT was incubated with varying concentrations of ligands for 2–4 h at 4 °C before incubating with the recombinant antibody fragment (Fab) 9D5 in a molar ratio of 1:1.2 (dDAT:Fab) for 30 mins on ice. The dDAT-9D5 complex was concentrated to a final concentration of 3.0–4.0 mg/ml using a 100 kDa cutoff centrifugal concentrator (Amicon Ultra). The concentrated sample was clarified to remove aggregates by high-speed centrifugation at 14,500 × *g* for 30 mins. The clarified sample was then subjected to crystallization by hanging-drop vapor diffusion method at 4 °C. Crystals of dDAT_subB_ and dDAT_NET_ proteins were obtained after 2–4 weeks, whereas those for dDAT_mfc_ were obtained in a week after seeding the crystallization drops with the dDAT_NET_ crystals. All crystals were obtained in 0.1 M MOPS, pH 6.5–7.0 and 30–32% PEG 600 as precipitant at 4 °C. Data from crystals were collected at different synchrotrons sources (Supplementary Table 1) and crystals for all data sets diffracted in a resolution range of 2.8–3.3 Å. The diffraction data were processed using XDS^61^ and merged and scaled using AIMLESS in the CCP4 software suite^62^. Five percent of the reflections were randomly assigned for *R*_free_ calculations as part of cross-validation. The structures were solved by molecular replacement through PHASER^63^ using the coordinates of dDAT and 9D5 from PDB ids 4XP1 or 4XNX. Refinement of the coordinates against the diffraction data were done using phenix.refine in the PHENIX crystallographic software suite^64^. Protein and inhibitor structures were built using COOT^65^ and modeling for lower resolution datasets was done with the aid of feature-enhanced map employed in the PHENIX suite^66^.

### DA transport and inhibition assays

Uptake of DA by the transport active constructs of *dDAT* (*dDAT*_*fc*_ and *dDAT*_*mfc*_) used in this study was performed in HEK293S GnTI^−^ cells transfected with the cGFP fused *dDAT* constructs. Cells were transiently transfected with pEG-BacMam plasmid harboring the *dDAT cGFP* gene and incubated for 35–40 hrs at 37 °C with 5% CO_2_ and 80% humidity. For the determination of inhibition potencies (IC_50_ values), transfected cells were resuspended in uptake assay buffer (25 mM HEPES-Tris pH 7.1, 130 mM NaCl, 5.4 mM KCl, 1.2 mM CaCl_2_, 1.2 mM MgSO_4_, 1 mM ascorbic acid, 5 mM glucose and 30 μM pargyline (Sigma Aldrich), a monoamine oxidase inhibitor) and incubated with varying concentrations of inhibitors at room temperature for 30 mins. This was followed by the addition of 2 μM of DA in a 1:100 molar ratio of [^3^H]-DA (Vitrax): [^1^H]-DA (Sigma Aldrich) and incubated for 20 mins at room temperature. The transport activity was arrested with 0.5 ml of pre-chilled assay buffer containing 100 μM desipramine (Sigma Aldrich) added to each reaction. The cells were then washed twice with the same buffer before solubilizing them in 0.1 ml of 20 mM DDM for one hour at room temperature. Post-solubilization the material was subjected to centrifugation at 14,500 × *g* for 30 mins to separate unsolubilized material. Following this, 0.1 ml of the supernatant was added to 0.5 ml of scintillation fluid (Ultima Gold, Perkin Elmer) and the radioactivity was estimated on MicroBeta scintillation counter (Perkin Elmer). Counts measured from cells incubated with 25 μM desipramine were considered as background. The background-subtracted, dose–response plots were analyzed using GraphPad Prism v.5.0.1 and *K*_*i*_ values were determined from Cheng–Prusoff’s equation using the IC_50_ values obtained from the experiments.

For the determination of Michaelis–Menten constant (*K*_*M*_), the *dDAT*_*fc*_ transfected HEK293S GnTI^-^ cells were incubated with varying concentrations of DA (0.2 μM, 0.4 μM, 2 μM, 4 μM, 8 μM, 10 μM, and 20 μM) in 1:200 molar ratio of [^3^H]-DA:[^1^H]-DA at room temperature in a 96-well plate. The uptake was arrested after 3 mins incubation at room temperature with 100 μM desipramine. The cells were washed twice with uptake buffer before solubilizing in 50 μl of 20 mM DDM. To this solubilized material 50 μl of scintillation fluid was added and the radioactivity was estimated on MicroBeta scintillation counter. The activity measured in untransfected cells was considered as background uptake. The background-subtracted initial uptake rates were plotted against above mentioned concentrations of DA to deduce the *K*_*M*_ value.

### Binding and competition assays

Binding assays were performed with 20 nM of purified dDAT protein by scintillation proximity assay. For determining nisoxetine *K*_*d*_, [^3^H] Nisoxetine (Perkin Elmer) (in 1:5 molar ratio) was used in the range of 0.1 nM to 500 nM with 100 µM desipramine added to the control samples. Competition assays were done with 50 nM [^3^H] Nisoxetine (in 1:5 molar ratio) with the concentration range for tramadol (Sigma Aldrich) being 100 nM to 3 mM, duloxetine (Sigma Aldrich) from 0.1 nM to 30 µM and milnacipran (Sigma Aldrich) from 1 nM to 100 µM. The assays were done in 1 mM DDM, 0.2 mM CHS, 20 mM Tris-Cl (pH 8), 300 mM NaCl and 5% glycerol. The background values were subtracted to plot the final curves in GraphPad prism v5.0.1 and the *K*_*d*_ values were calculated. The IC_50_ values obtained from binding competition assays were used to deduce the *K*_*i*_ values by Cheng–Prusoff’s equation.

### Statistical analysis

The biochemical assays performed were analyzed using GraphPad Prism v5.0.1. The significance tests were performed using two-sample *t* test assuming unequal variances in Microsoft Excel.

### Reporting summary

Further information on research design is available in the Nature Research Reporting Summary linked to this article.

## Supplementary information

Supplementary Information

Description of Additional Supplementary Files

Supplementary Movie 1

Reporting Summary

## Data Availability

Data supporting the findings of this manuscript are available from the corresponding author upon reasonable request. A reporting summary for this Article is available as a Supplementary Information file. The coordinates for the structures have been deposited in the Protein Data Bank with the following accession codes PDB6M0F, PDB6M0Z, PDB6M2R, PDB6M38, PDB6M3Z, PDB6M47. Source data are provided with this paper.

## Source data

Source Data
