# Supplementary material for: Structural basis of norepinephrine recognition and transport inhibition in neurotransmitter transporters
Source: Nat Commun. 2021 Apr 13;12:2199. doi: 10.1038/s41467-021-22385-9 (PMC8044178; doi:10.1038/s41467-021-22385-9)
Supplement: Supplementary file 1 — Supplementary Information [file 41467_2021_22385_MOESM1_ESM.pdf]

Supplementary Information

Structural basis of norepinephrine recognition and transport inhibition in neurotransmitter transporters

Shabareesh Pidathala[1], Aditya Kumar Mallela[1], Deepthi Joseph[1] and Aravind Penmatsa[1,*]

[1]Molecular Biophysics Unit, Indian Institute of Science, CV Raman Road, Bangalore 560012.

Running Head. Norepinephrine and inhibitor recognition in neurotransmitter transporters

*Corresponding author. Correspondence and requests for material may be addressed to AP (Email. penmatsa@iisc.ac.in; Phone. +91-8022932458)

Supplementary Figures 1-8
Supplementary Tables 1-3

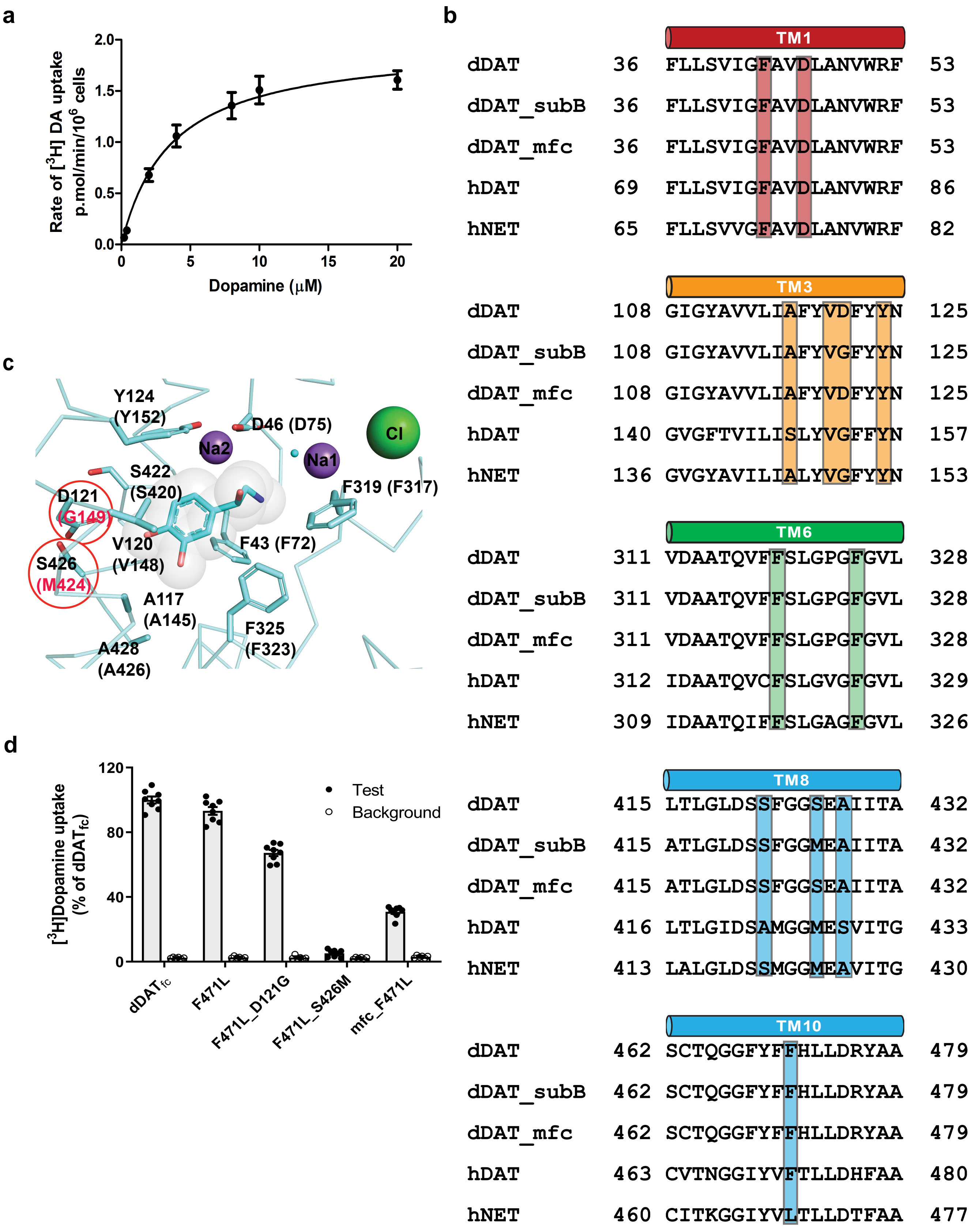

Supplementary Fig. 1. a, [3H] Dopamine uptake kinetics of dDAT functional construct with F471L mutation. The plot is an average of n = 6 measurements obtained over 2 independent experiments with error bars representing s.e.m. K_M value measured was 3.6 ± 0.8 μM. **b,** Multiple sequence alignment of the helices surrounding the primary substrate/inhibitor binding site with residues that sculpt the binding pocket highlighted as vertical bars. **c,** Binding site residues and subsite B mutants performed in the study represented in the structure with bound L-norepinephrine. The corresponding amino-acids of hNET are indicated in parenthesis. **d,** [3H] Dopamine uptake analysis using subsite B mutants D121G and S426M in the context of F471L mutant. S426M causes a loss of transport activity. The plot is an average of n = 8 measurements obtained over 3 independent experiments with error bars representing s.e.m. Source data are provided as a Source Data file.

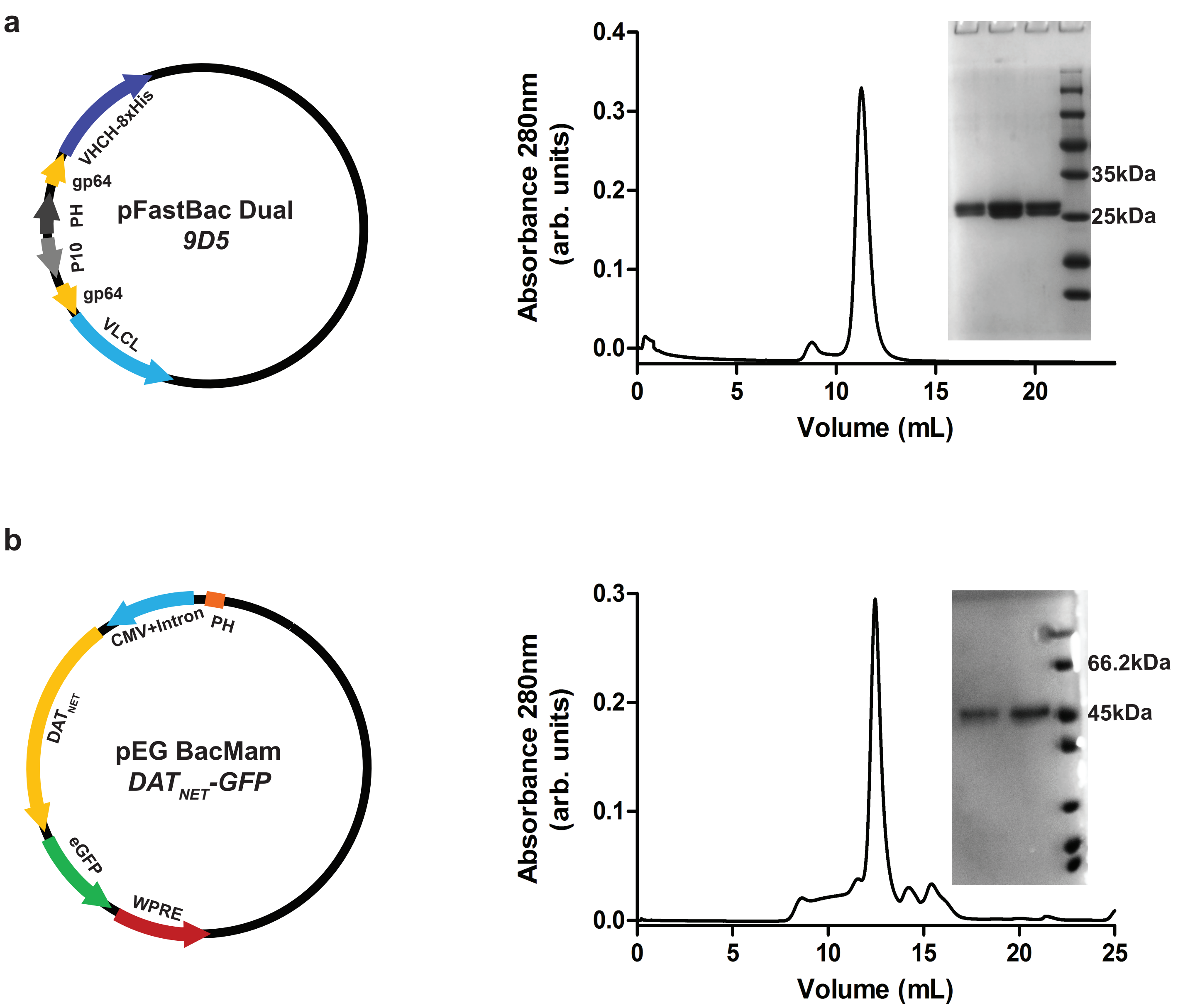

Supplementary Fig. 2. **a,** Vector design of pFastBac dual containing the heavy and light chains of the *9D5* antibody fragment cloned downstream of the polyhedrin (PH) and p10 promoters, respectively. Both genes were cloned with a gp64 signal peptide at the N-terminus to facilitate export from insect cells. Panel on the right shows a representative size exclusion chromatogram of the purified Fab and inset displays heavy and light chain bands for the purified 9D5 with coomassie stained SDS-PAGE (12%). The protein purification was repeated 20 times with consistant results. **b,** Vector design of the *dDAT* constructs in pEG BacMam with a C-terminal *GFP* that was cleaved by thrombin cleavage post-expression. Panel on right shows a representative chromatogram of purified dDAT and the corresponding protein band stained with coomassie on an SDS-PAGE (12%). The protein purification was repeated 20 times with consistant results.

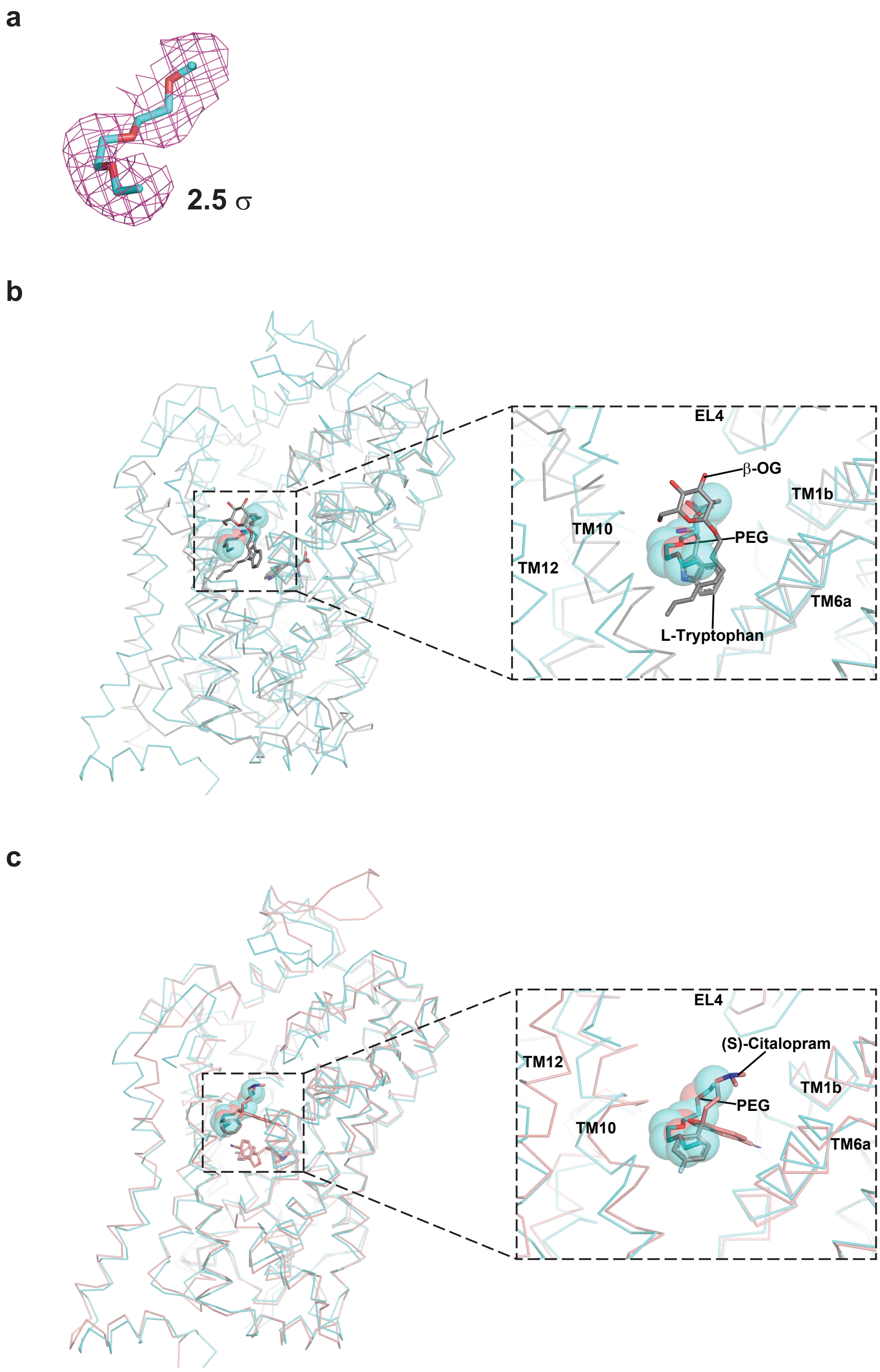

Supplementary Fig. 3. a, The *Fo-Fc* map of PEG (2.5 σ) observed in the secondary binding site of $dDAT_{subB}$ in its substrate free state (apo). **b,** Overlay of $dDAT_{apo}$ with LeuT (PDB id. 3F3A) with inset showing bound n-octyl-β-D-glucopyranoside (β-OG) and L-tryptophan in LeuT and PEG in $dDAT_{apo}$ in the extracellular vestibule. **c,** Overlay of $dDAT_{apo}$ with hSERT (PDB id. 5I73) with inset showing (S)-citalopram in hSERT and PEG in $dDAT_{apo}$ in the extracellular vestibule.

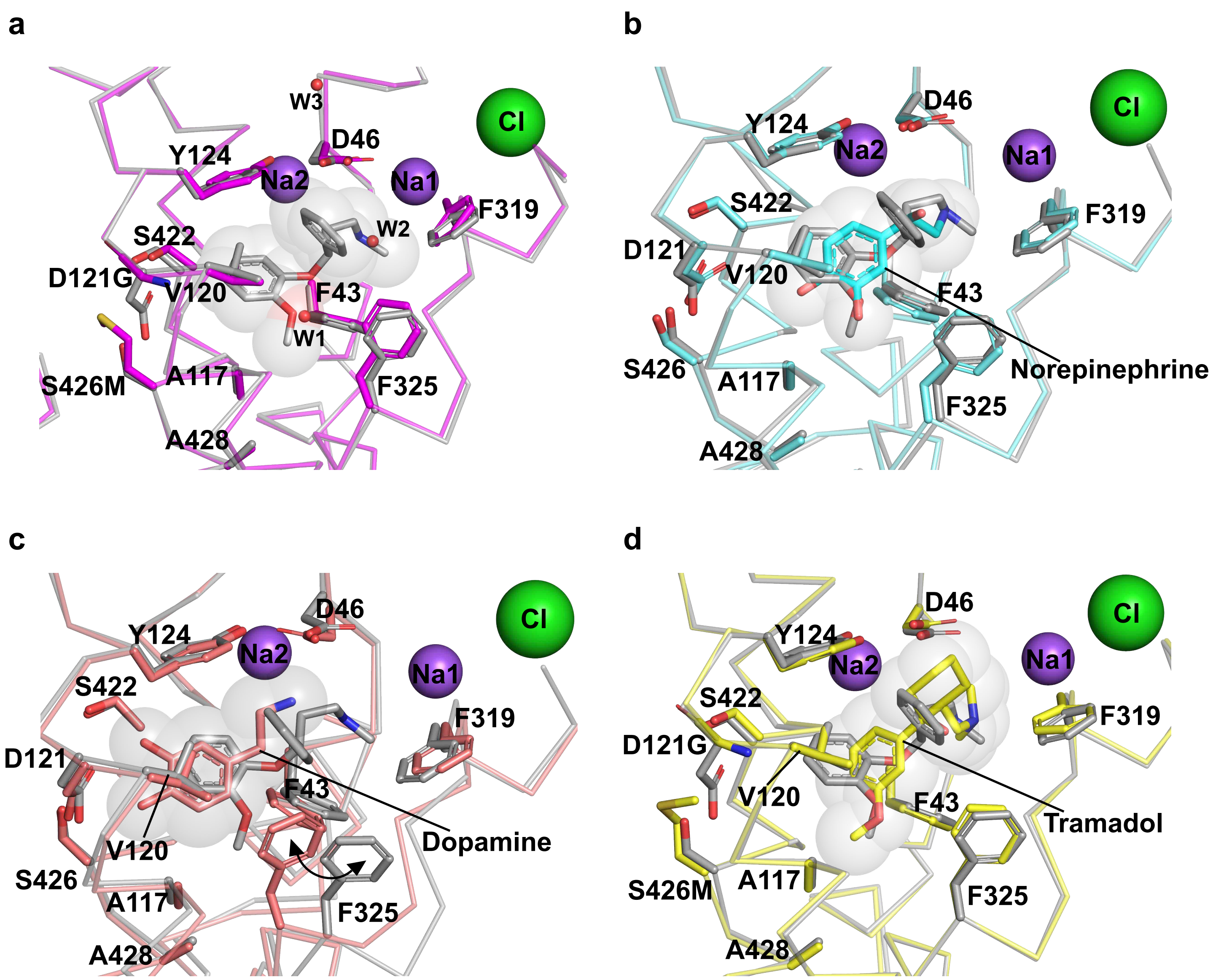

Supplementary Fig. 4. a, Overlay of nisoxetine bound dDAT (gray) (4XNU) with substrate-free conformation of $\mathrm{dDAT_{subB}}$ to highlight differences in the binding pocket. Similar structural comparisons with **b,** L-norepinephrine bound $\mathrm{dDAT_{mfc}}$, **c,** dopamine bound $\mathrm{dDAT_{mfc}}$, **d,** tramadol bound $\mathrm{dDAT_{NET}}$ display residues that interact with the inhibitors in the primary binding site and shifts in their positions.

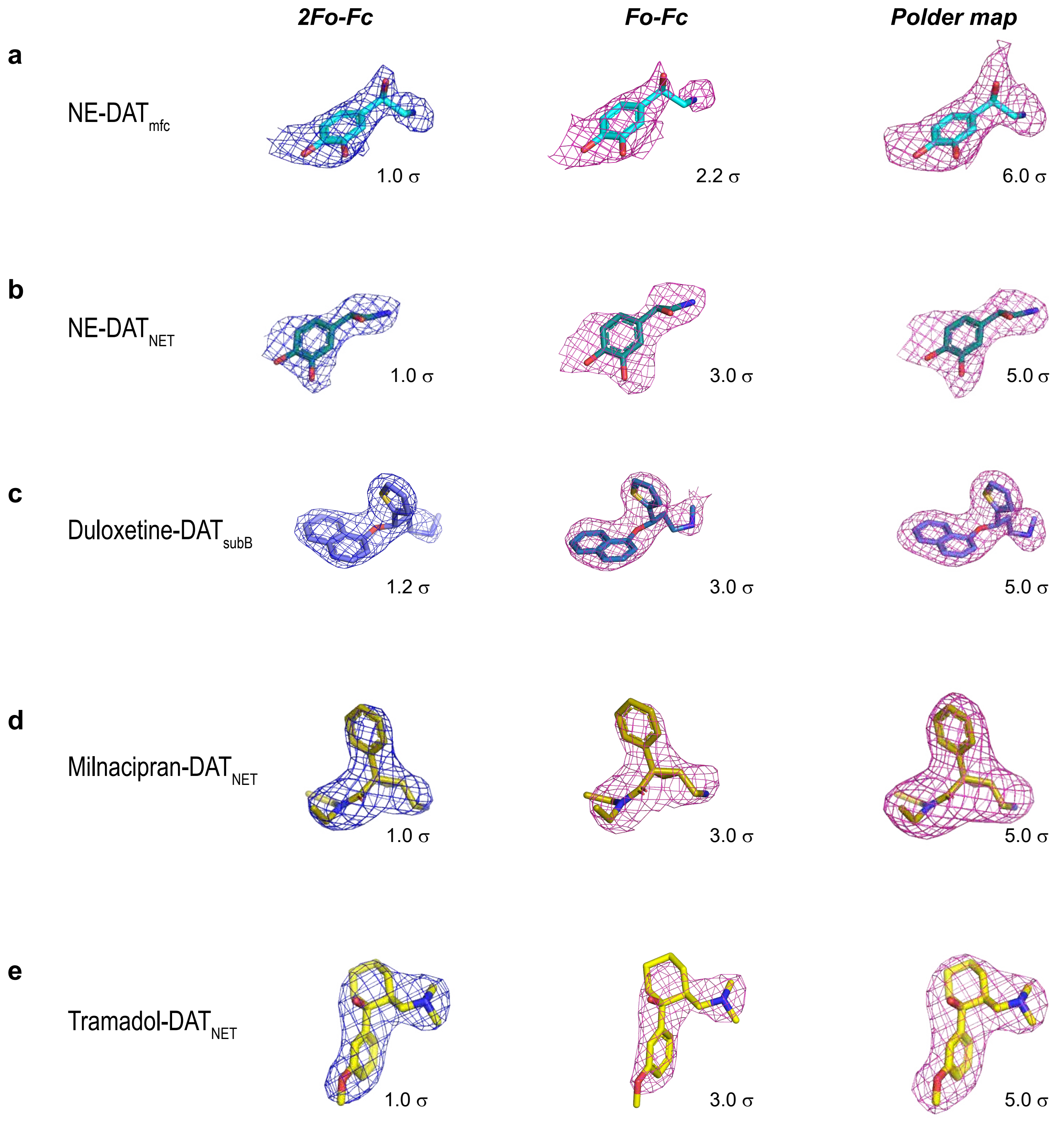

Supplementary Fig. 5. Experimental electron densities of substrate and inhbitors bound in the binding pocket of dDAT structures in this study. Column 1 represents 2*Fo-Fc* maps (blue), column 2 represents *Fo-Fc* omit maps (magenta) and column 3 represents Polder maps (magenta) for **a,** NE bound $dDAT_{mfc}$ **b,** NE bound $dDAT_{NET}$ **c,** duloxetine bound $dDAT_{subB}$ **d,** milnacipran bound $dDAT_{NET}$ **e,** tramadol bound $dDAT_{NET}$

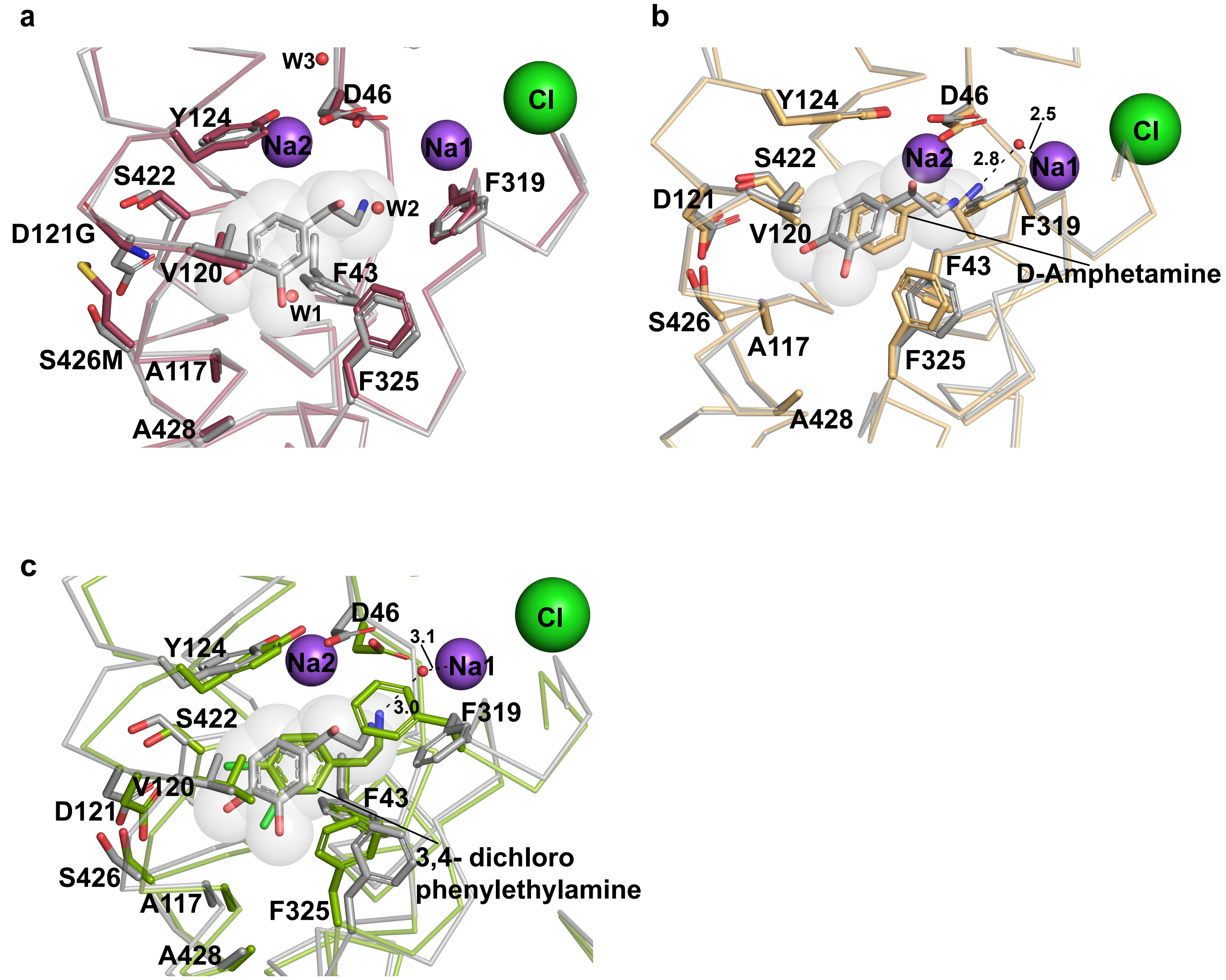

Supplementary Fig. 6. Structural comparisons between NE bound $dDAT_{mfc}$ (gray) with **a,** substrate free state of dDAT, **b,** D-amphetamine bound $dDAT_{mfc}$ (PDB id 4XP9), **c,** 3,4 dichlorophenethylamine bound $dDAT_{mfc}$ (PDB id 4XPA).

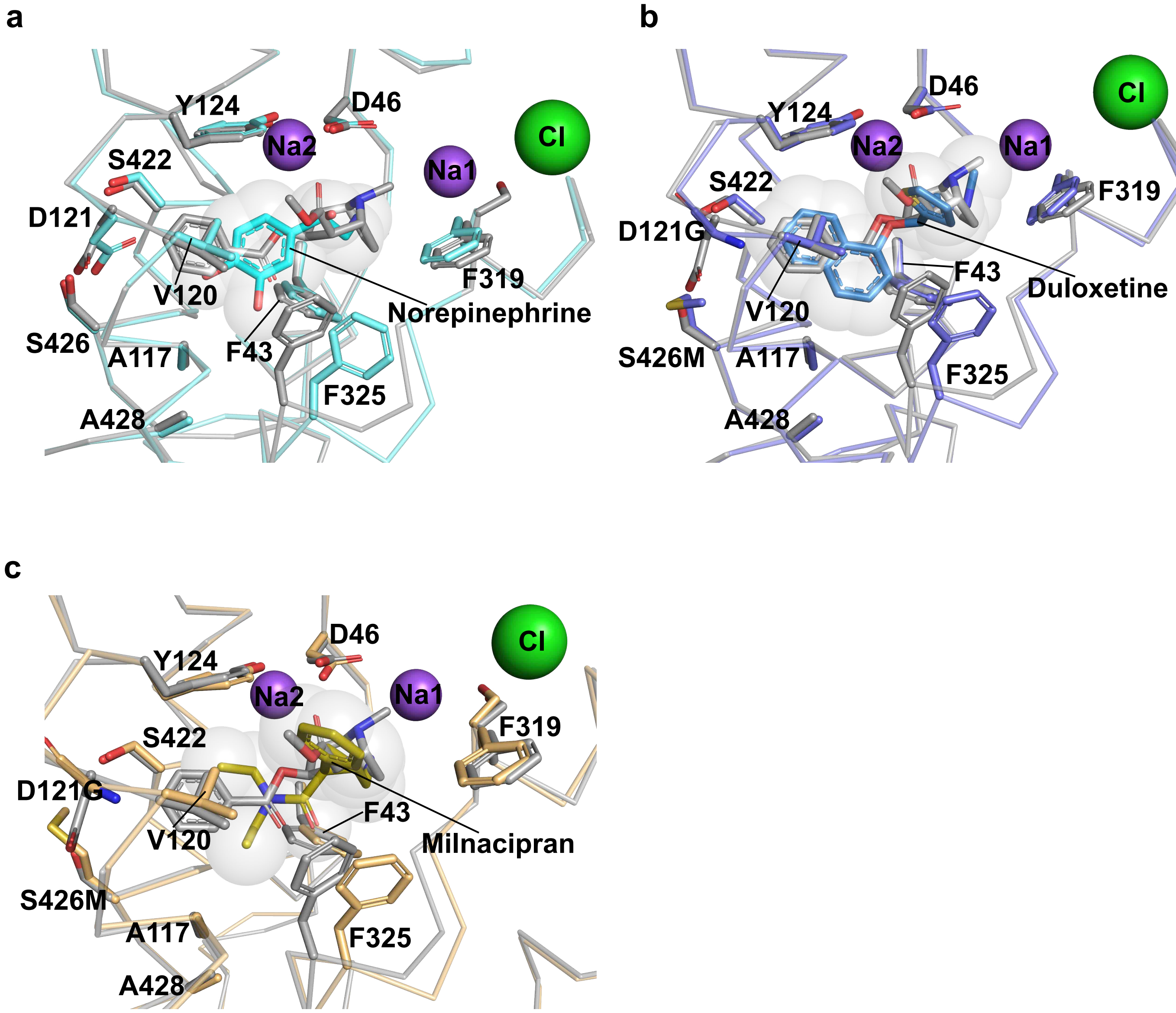

Supplementary Fig. 7. Structural overlays between cocaine bound $dDAT_{mfc}$ (gray) (PDB id. 4XP4) with **a,** NE bound $dDAT_{mfc}$, **b,** duloxetine bound $dDAT_{subB}$, **c,** milnacipran bound $dDAT_{NET}$ to display changes in residue positions within the primary binding site.

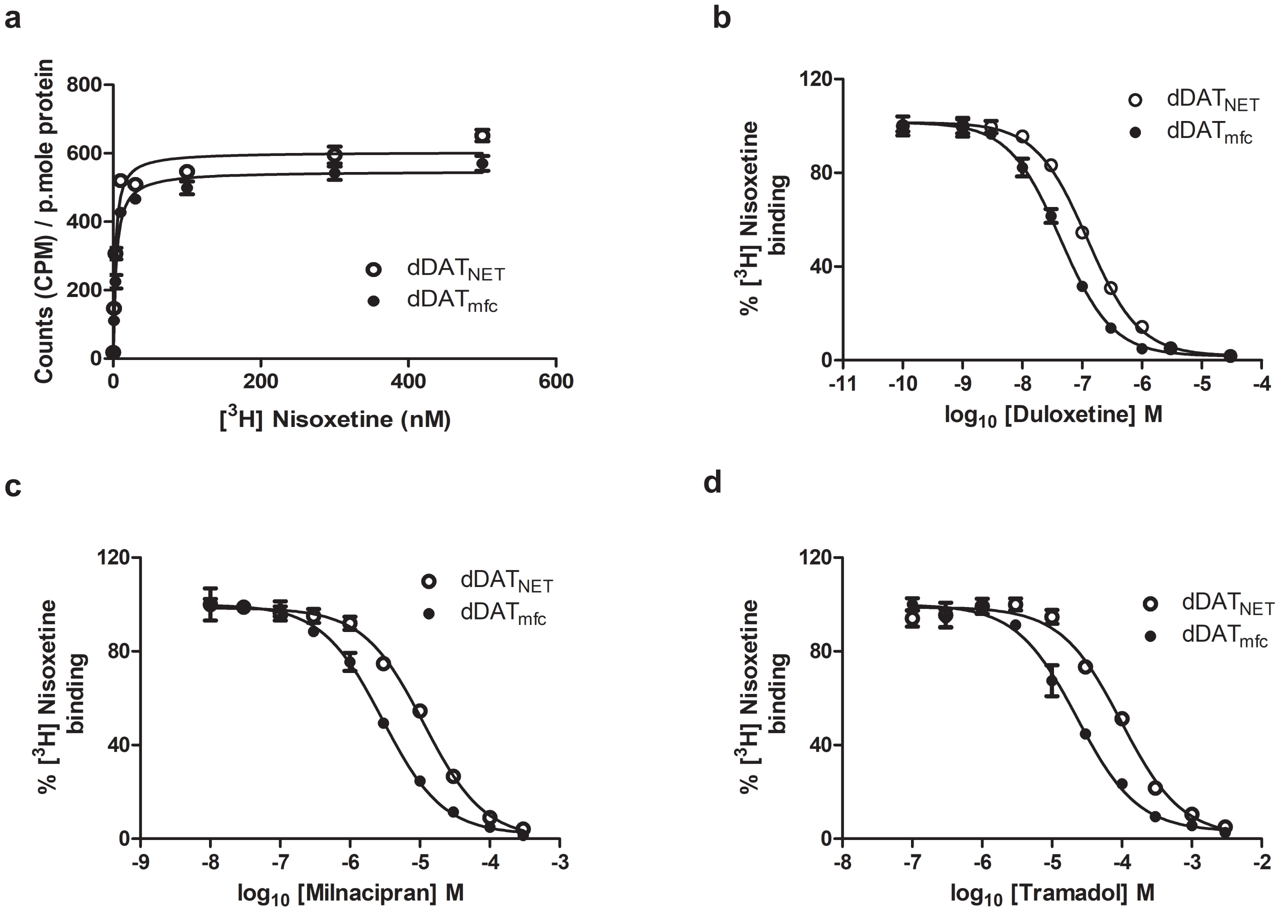

NET inhibitor	Binding K_i with $dDAT_{NET}$	Binding K_i with $dDAT_{mfc}$
**Duloxetine**	6.52 ± 0.52 nM	3.18 ± 0.31 nM
**Milnacipran**	605.18 ± 25.28 nM	214.8 ± 13.82 nM
**Tramadol**	5.31 ± 0.45 μM	1.89 ± 0.16 μM

Supplementary Fig. 8. a, Binding curves of [^{3}H] nisoxetine with $dDAT_{mfc}$ and $dDAT_{NET}$ containing mutations in primary binding site D121G and S426M. The plot is an average of 9 measurements with error bars representing s.e.m. K_d values of 3.82 ± 0.39 nM and 2.79 ± 0.27 nM were measured for $dDAT_{mfc}$ and $dDAT_{NET}$ respectively. Competitive inhibition of [^{3}H] nisoxetine binding with increasing concentrations of **b,** duloxetine, **c,** milnacipran , **d,** tramadol performed for $dDAT_{mfc}$ (solid circles) and $dDAT_{NET}$ (open circles). All plots are an average of 9 measurements each with error bars representing s.e.m. K_i values reported in the table below were calculated from estimated IC_{50} values of each inhibition curve and K_d values estimated from nisoxetine binding using the Cheng-Prusoff equation. Each plot is an average of n = 9 measurements obtained over 3 independent experiments with error bars representing s.e.m. Source data are provided as a Source Data file.

Supplementary Table 1. Data collection and refinement statistics

Parameter	Substrate-free $dDAT_{subB}$	NE-$dDAT_{mfc}$	NE-$dDAT_{NET}$	S-duloxetine $dDAT_{subB}$	Milnacipran $dDAT_{NET}$	Tramadol $dDAT_{NET}$
Source	ESRF-ID29	ElettraXRD2	APS-24IDC	ElettraXRD2	APS-24IDC	SLS-PX1
Wavelength	0.968	0.979	0.979	0.979	0.979	1.000
Space group	$P2_12_12_1$	$P2_12_12_1$	$P2_12_12_1$	$P2_12_12_1$	$P2_12_12_1$	$P2_12_12_1$
Cell dimensions *a, b, c* (Å); $\alpha = \beta = \gamma = 90°$	97.7, 133.3, 162.0	96.8, 141.2, 167.6	97.1, 142.5, 168.0	97.3, 140.6, 167.8	96.5, 140.9, 167.3	97.0, 140.9, 167.8
Resolution (Å), (*)	50.0-3.3 (3.5-3.3)	50.0-2.8 (2.97-2.8)	50.0-2.88 (3.05-2.88)	50.0-3.0 (3.18-3.0)	50.0-3.11 (3.29-3.11)	50.0-3.25 (3.45-3.25)
Total Observations	180963 (27837)	352419 (55239)	358042 (55889)	338525 (54597)	228053 (37703)	193872 (32191)
Unique reflections	32633 (5148)	109219 (17635)	53971 (8591)	88712 (14205)	42395 (6747)	64838 (10759)
Multiplicity	5.5 (5.4)	3.22 (3.13)	6.6 (6.5)	3.81(3.84)	5.4(5.6)	3.0 (3.0)
Completion (%)	99.7(99.0)	99.5 (98.9)	99.9 (99.9)	99.6 (99.0)	99.6(99.3)	93 (95.8)
$I/\sigma I$	10.6(0.94)	11.4 (1.1)	13.4(1.0)	11.3 (1.2)	7.64(1.0)	8.39 (1.0)
$CC_{1/2}$ (%)	99.9(60.9)	99.8 (53.6)	99.8 (43.3)	99.8 (49.3)	99.7 (47.5)	99.9 (84.1)
R_{meas} (%)	11.6 (148.9)	10.3 (119.0)	10.2 (206.6)	10.7 (116.2)	17.4 (135.4)	15.1 (132.3)
Refinement Statistics						
Resolution	50.0-3.3	50.0-2.8	50.0-2.88	50.0-3.0	50.0-3.1	50.0-3.25
Reflections[#]	32557 (3144)	57114 (5587)	53940 (5335)	46691 (4542)	42341 (4157)	35194 (3543)
R_{work} (%)	24.3 (37.0)	21.2 (34.3)	21.2 (34.0)	21.5 (30.9)	22.3 (32.3)	24.2 (32.2)
R_{free} (%)	27.6 (40.5)	24.8 (39.0)	24.7 (36.4)	24.4 (32.5)	26.5 (36.8)	27.8 (37.3)
No. of non-H atoms	7508	7780	7654	7634	7670	7615
Protein	7430	7538	7507	7490	7504	7455
Substrate/Inhibitor	-	12	12	21	18	19
Ions/Lipid/Detergent	70	131	99	99	128	122
water	8	99	36	24	20	19
Average B-factor	112.9	64.2	89.92	78.3	84.6	89.4
Protein	112.9	64.05	89.73	78.2	84.4	89.1
Substrate/Inhibitor	-	87.8	102	71.5	79.7	88.0
Detergent/Lipid/PEG	117.0	77.4	101	89	97	92.5
water	92.3	60.1	92.7	69.9	75.8	80.1
RMS bond lengths (Å)	0.004	0.006	0.005	0.006	0.01	0.005
RMS bond angles (º)	1.01	1.12	1.00	1.1	1.40	1.17
Ramachandran statistics						
Favoured	92.3	94.60	94.70	95.8	94.8	95.1
Allowed	7.7	5.4	5.3	4.2	5.2	4.9
Disallowed	0	0	0	0	0	0

*** Values in parentheses represent highest-resolution shell.**

[#] Reflections employed in structure refinement.

Supplementary Table 2.

a Uptake Inhibition

	**Duloxetine**	**Milnacipran**	**Tramadol**
**hNET**	K_i = 81 ± 19 nM[1]	K_i = 52 ± 9 nM[1]	K_i = 19.7 µM[2]
**$dDAT_{fc}$**	K_i = 69.4 ± 2.9 nM (This study)	K_i = 1.5 ± 0.1 µM (This study)	K_i = 15.9 ± 1.8 µM (This study)
**hDAT**	K_i = 370 nM[3]	K_i = >100,000 nM[4]	IC_{50} = 100 µM[5]<br>K_i = N/D

b Binding Inhibition (K_i)

	**Duloxetine**	**Milnacipran**	**Tramadol**
**hNET**	1.17 ± 0.11 nM (vs. Nisoxetine)[4]	22 ± 2.58 nM (vs. Nisoxetine)[4]	11.2 µM (vs. desipramine)[2]
**$dDAT_{NET}$**	6.52 ± 0.52 nM (vs. Nisoxetine)<br>(This study)	605.18 ± 25.28 nM (vs. Nisoxetine)<br>(This study)	5.31 ± 0.45 µM (vs. Nisoxetine)<br>(This study)
**hDAT**	230 ± 17 nM<br>(vs. RTI-55)[4]	>100,000 nM<br>(vs. RTI-55)[4]	N/D

c Nisoxetine binding affinity

	**$dDAT_{NET}$**	**$dDAT_{mfc}$**	**hNET**	**hDAT**
**Nisoxetine K_d (nM)**	2.79 ± 0.27 (This study)	3.82 ± 0.39 (This study)	5.1[6]	N/D

Supplementary References

1. Sorensen, L. et al. Interaction of antidepressants with the serotonin and norepinephrine transporters: mutational studies of the S1 substrate binding pocket. *J Biol Chem* **287**, 43694-707 (2012).
2. Sagata, K. et al. Tramadol inhibits norepinephrine transporter function at desipramine-binding sites in cultured bovine adrenal medullary cells. *Anesth Analg* **94**, 901-6, (2002).
3. Bymaster, F.P. et al. Duloxetine (Cymbalta), a dual inhibitor of serotonin and norepinephrine reuptake. *Bioorg Med Chem Lett* **13**, 4477-80 (2003).
4. Vaishnavi, S.N. et al. Milnacipran: a comparative analysis of human monoamine uptake and transporter binding affinity. *Biol Psychiatry* **55**, 320-2 (2004).
5. Rickli, A., Liakoni, E., Hoener, M.C. & Liechti, M.E. Opioid-induced inhibition of the human 5-HT and noradrenaline transporters in vitro: link to clinical reports of serotonin syndrome. *Br J Pharmacol* **175**, 532-543 (2018).
6. Paczkowski, F.A., Sharpe, I.A., Dutertre, S. & Lewis, R.J. chi-Conotoxin and tricyclic antidepressant interactions at the norepinephrine transporter define a new transporter model. *J Biol Chem* **282**, 17837-44 (2007).

Supplementary Table 3

Primer	Sequence
A117S_FP	5’GTGGTGCTGATATCCTTCTATGTGGACTT3’
D121G_FP	5’GCCTTCTATGTGGGCTTCTATTACAATG3’
S426M_FP	5’GTTCGTTTGGTGGTATGGAGGCTATAATC3’
A428S_RP	5’AGCTGTGATTATAGACTCTGAACCACC3’
F471L_FP	5’GGTGGCTTCTATTTCTTGCATCTGCTGGATCGT3’
9D5Hc_RP	5’GACAAGCTTTCAGTGATGGTGGTGATGATGATGGTGGGCGGC CGCTGATTGGAAGTACAGGTTTTC3’
9D5Hc_FP	5’GCGGAATTCGCCACCATGGTAAGCGCTATTGTTTTATATG3’
9D5Lc_RP	5’CTGCTAGCCTAGCATTCGTTGCGGTTAAAAC3’
9D5Lc_RP	5’GATCTCGAGGCCACCATGGTAAGCGCTATTGTTTTATATGTG3’
