# Supplementary material for: Structural basis of norepinephrine recognition and transport inhibition in neurotransmitter transporters
Source: Nat Commun. 2021 Apr 13;12:2199. doi: 10.1038/s41467-021-22385-9 (PMC8044178; doi:10.1038/s41467-021-22385-9)
Supplement: Supplementary file 2 — Description of Additional Supplementary Files [file 41467_2021_22385_MOESM2_ESM.docx]

**Description of Additional Supplementary Files**

File Name: Supplementary Movie 1

Description: The structure of norepinephrine bound dDATmfc morphed over the structure of dopamine bound dDATmfc (PDB id: 4XP1) highlighting the structural changes in the substrate bind pocket. The TM6 linker undergoes an inward movement by 2.4 Å in the dopamine bound state relative to the norepinephrine bound state. This inward movement positions the F325 side-chain closer to dopamine, which is bound in proximity to subsite B relative to norepinephrine, which is bound closer to subsite C. This structural change is accompanied by ~260° flip in the χ1 torsional angle of D46 side chain and a 16° χ2 torsion angle shift in the side chain of D121.
