# Supplementary material for: Structural basis of norepinephrine recognition and transport inhibition in neurotransmitter transporters
Source: Nat Commun. 2021 Apr 13;12:2199. doi: 10.1038/s41467-021-22385-9 (PMC8044178; doi:10.1038/s41467-021-22385-9)
Supplement: Supplementary file 4 — Reporting Summary [file 41467_2021_22385_MOESM4_ESM.pdf]

nature research

Corresponding author(s): Aravind Penmatsa

Last updated by author(s): 2021/02/11

Reporting Summary

Nature Research wishes to improve the reproducibility of the work that we publish. This form provides structure for consistency and transparency in reporting. For further information on Nature Research policies, see our Editorial Policies and the Editorial Policy Checklist.

Statistics

For all statistical analyses, confirm that the following items are present in the figure legend, table legend, main text, or Methods section.

n/a	Confirmed	
☐	☒	The exact sample size (*n*) for each experimental group/condition, given as a discrete number and unit of measurement
☐	☒	A statement on whether measurements were taken from distinct samples or whether the same sample was measured repeatedly
☐	☒	The statistical test(s) used AND whether they are one- or two-sided<br>*Only common tests should be described solely by name; describe more complex techniques in the Methods section.*
☒	☐	A description of all covariates tested
☒	☐	A description of any assumptions or corrections, such as tests of normality and adjustment for multiple comparisons
☐	☒	A full description of the statistical parameters including central tendency (e.g. means) or other basic estimates (e.g. regression coefficient) AND variation (e.g. standard deviation) or associated estimates of uncertainty (e.g. confidence intervals)
☐	☒	For null hypothesis testing, the test statistic (e.g. *F*, *t*, *r*) with confidence intervals, effect sizes, degrees of freedom and *P* value noted<br>*Give P values as exact values whenever suitable.*
☒	☐	For Bayesian analysis, information on the choice of priors and Markov chain Monte Carlo settings
☒	☐	For hierarchical and complex designs, identification of the appropriate level for tests and full reporting of outcomes
☒	☐	Estimates of effect sizes (e.g. Cohen's *d*, Pearson's *r*), indicating how they were calculated

Our web collection on statistics for biologists contains articles on many of the points above.

Software and code

Policy information about availability of computer code

Data collection	Crystallographic data were collected through the mxcube software (ESRF/Elettra) or RAPD software (APS/NECAT) using Pilatus 6M detectors
Data analysis	Graphpad Prism version 5.0.1, Microsoft Excel version 16.16.8, Phenix1.13-2998, Coot0.8.9.1, XDS vMarch15-2019, CCP4-7.0.067, PHASER (implemented as part of Phenix suite)

For manuscripts utilizing custom algorithms or software that are central to the research but not yet described in published literature, software must be made available to editors and reviewers. We strongly encourage code deposition in a community repository (e.g. GitHub). See the Nature Research guidelines for submitting code & software for further information.

Data

Policy information about availability of data

All manuscripts must include a data availability statement. This statement should provide the following information, where applicable:

- Accession codes, unique identifiers, or web links for publicly available datasets
- A list of figures that have associated raw data
- A description of any restrictions on data availability

The coordinates for the determined structures were deposited in the Protein Data Bank with the following accession codes 6M0F, 6M0Z, 6M2R, 6M38, 6M3Z and 6M47. Source Data generated as part of the study will be made available as supplementary information.

Field-specific reporting

Please select the one below that is the best fit for your research. If you are not sure, read the appropriate sections before making your selection.

☒ Life sciences ☐ Behavioural & social sciences ☐ Ecological, evolutionary & environmental sciences

For a reference copy of the document with all sections, see nature.com/documents/nr-reporting-summary-flat.pdf

Life sciences study design

All studies must disclose on these points even when the disclosure is negative.

Sample size	All biochemical experiments were done twice or thrice as independent sets. Proteins for structural and binding studies were purified multiple times and were found to retain identical biochemical and crystallization properties.
Data exclusions	No data was excluded from analysis
Replication	All the biochemical studies were performed in triplicates repeated twice or thrice as mentioned in the figure legends. For structural studies, protein purification was performed multiple times and the purified protein displayed identical biochemical and crystallographic properties. All replication attempts were successful.
Randomization	During structure refinement, 5% of crystallographic reflections were randomly selected and kept out for cross validation.
Blinding	In structural studies, the randomly selected set of reflections served as an internal control for the quality of final structures.

Reporting for specific materials, systems and methods

We require information from authors about some types of materials, experimental systems and methods used in many studies. Here, indicate whether each material, system or method listed is relevant to your study. If you are not sure if a list item applies to your research, read the appropriate section before selecting a response.

Materials & experimental systems

n/a	Involved in the study	
☐	☒	Antibodies
☐	☒	Eukaryotic cell lines
☒	☐	Palaeontology and archaeology
☒	☐	Animals and other organisms
☒	☐	Human research participants
☒	☐	Clinical data
☒	☐	Dual use research of concern

Methods

n/a	Involved in the study	
☒	☐	ChIP-seq
☒	☐	Flow cytometry
☒	☐	MRI-based neuroimaging

Antibodies

Antibodies used	The antibody fragment (Fab) used in the study is a recombinant version of the same antibody used in the following study https://www.nature.com/articles/nature12533/. The process of recombinant production is explained in the Methods section of the manuscript.
Validation	The species specific validation of Fab was performed in the following study https://www.nature.com/articles/nature12533/. We have generated the recombinant version of the same Fab in insect cells. The recombinant Fab was validated for its ability to bind the target protein by looking for shifts in the chromatogram peaks of Fab bound and unbound protein. The purity and homogeneity of the Fab was validated by size exclusion chromatography and SDS-PAGE as shown in extended figure 2B of the manuscript. We also validated the purity of recombinant Fab by ESI-MS followed by structure elucidation of dDAT-Fab complexes.

Eukaryotic cell lines

Policy information about cell lines

Cell line source(s)	HEK293SGnTI- from ATCC. Sf9 cell line from Invitrogen
Authentication	The cell lines were not authenticated by us.
Mycoplasma contamination	The cell lines were not tested for Mycoplasma contamination.
Commonly misidentified lines (See ICLAC register)	No commonly misidentified cell lines were used in the study.
